# Global Profiling of Lysine Acetylation in *Borrelia burgdorferi* B31 Reveals Its Role in Central Metabolism

**DOI:** 10.3389/fmicb.2018.02036

**Published:** 2018-08-31

**Authors:** Sébastien Bontemps-Gallo, Charlotte Gaviard, Crystal L. Richards, Takfarinas Kentache, Sandra J. Raffel, Kevin A. Lawrence, Joseph C. Schindler, Joseph Lovelace, Daniel P. Dulebohn, Robert G. Cluss, Julie Hardouin, Frank C. Gherardini

**Affiliations:** ^1^Laboratory of Bacteriology, Rocky Mountain Laboratories, National Institute of Allergy and Infectious Diseases, National Institutes of Health, Hamilton, MT, United States; ^2^CNRS UMR 6270 Polymères, Biopolymères, Surfaces Laboratory, Université de Rouen, Mont-Saint-Aignan, France; ^3^PISSARO Proteomic Facility, Institut de Recherche et d’Innovation Biomédicale, Mont-Saint-Aignan, France; ^4^Department of Chemistry and Biochemistry, Middlebury College, Middlebury, VT, United States

**Keywords:** Lyme disease, *Borrelia*, acetylation, metabolism, regulation-post-translational

## Abstract

The post-translational modification of proteins has been shown to be extremely important in prokaryotes. Using a highly sensitive mass spectrometry-based proteomics approach, we have characterized the acetylome of *B. burgdorferi*. As previously reported for other bacteria, a relatively low number (5%) of the potential genome-encoded proteins of *B. burgdorferi* were acetylated. Of these, the vast majority were involved in central metabolism and cellular information processing (transcription, translation, etc.). Interestingly, these critical cell functions were targeted during both ML (mid-log) and S (stationary) phases of growth. However, acetylation of target proteins in ML phase was limited to single lysine residues while these same proteins were acetylated at multiple sites during S phase. To determine the acetyl donor in *B. burgdorferi*, we used mutants that targeted the sole acetate metabolic/anabolic pathway in *B. burgdorferi* (lipid I synthesis). *B. burgdorferi* strains B31-A3, B31-A3 Δ*ackA* (acetyl-P^-^ and acetyl-CoA^-^) and B31-A3 Δ*pta* (acetyl-P^+^ and acetyl-CoA^-^) were grown to S phase and the acetylation profiles were analyzed. While only two proteins were acetylated in the Δ*ackA* mutant, 140 proteins were acetylated in the Δ*pta* mutant suggesting that acetyl-P was the primary acetyl donor in *B. burgdorferi*. Using specific enzymatic assays, we were able to demonstrate that hyperacetylation of proteins in S phase appeared to play a role in decreasing the enzymatic activity of at least two glycolytic proteins. Currently, we hypothesize that acetylation is used to modulate enzyme activities during different stages of growth. This strategy would allow the bacteria to post-translationally stimulate the activity of key glycolytic enzymes by deacetylation rather than expending excessive energy synthesizing new proteins. This would be an appealing, low-energy strategy for a bacterium with limited metabolic capabilities. Future work focuses on identifying potential protein deacetylase(s) to complete our understanding of this important biological process.

## Introduction

*Borrelia burgdorferi* (also named *Borreliella burgdorferi*) (Barbour et al., 2017), the etiologic agent of Lyme disease, is transmitted to mammalian hosts through the bite of a hard *Ixodes* tick (Burgdorfer et al., 1982; Steere et al., 1983). Spirochetes are acquired by larval ticks during feeding on an infected vertebrate host. After feeding, larvae then molt into nymphs and bacteria can be transmitted to a mammalian host during the subsequent blood meal (Stewart and Rosa, 2017). In addition to overcoming the immune system of the host, the spirochetes also have to survive and adapt to extreme life conditions (e.g., starvation) in the midgut of the tick between feedings (Cabello et al., 2017; Stewart and Rosa, 2017). Understanding the metabolic adaptation of *B. burgdorferi* during the long-term survival is an essential step to understand this disease.

A successful infection relies on the ability to reach a Pareto optimality in metabolism to support growth sufficient for effective transition to a new host (Shoval et al., 2012; Angione et al., 2013; Szekely et al., 2013; Kafri et al., 2016; Lalanne et al., 2018). This optimal allocation of available resources during the early stage of feeding depends on ATP production but also on NADH/NAD^+^ balance (Snoep et al., 1994; de Graef et al., 1999; Henry et al., 2007; Sun et al., 2012; Bhat et al., 2016; Adams et al., 2017). *B. burgdorferi* has a limited metabolic capacity (Fraser et al., 1997; Casjens et al., 2000) which depends on the utilization of a few simple sugars and three carbon metabolites (e.g., glucose, glycerol, etc.) that are fermented to lactate via a chimeric Embden-Meyerhof pathway (von Lackum and Stevenson, 2005; Gherardini et al., 2010; Caimano et al., 2016; Troy et al., 2016). With no biochemical pathways for the *de novo* synthesis of the precursors that are required for DNA, RNA, fatty acid, cell wall, protein or lipid biosynthesis, *B. burgdorferi* expends energy to transport these molecules and assemble them into cellular macromolecules that are required for growth. Furthermore, with a slow growth rate, slow protein turnover (Gilbert et al., 2007; Srivastava and de Silva, 2008), a small genome, and relatively stable RNA (RNA half-life ∼45 min), these spirochetes have evolved to maximize the use of the ATP they generated from central metabolism (Archambault et al., 2013a,b).

Post-translational modifications (PTMs) of proteins are well described to rapidly respond to changing environmental conditions at a low energy cost (Cain et al., 2014). Reversible PTMs (e.g., phosphorylation, acetylation, succinylation) are better candidates for signaling and regulation than irreversible PTMs (e.g., glycosylation). In *B. burgdorferi*, the phosphorylation of Rrp1 affects the levels of the secondary messenger, cyclic di-GMP (c-di-GMP), (Rogers et al., 2009; Caimano et al., 2015) while the phosphorylation of Rrp2 up-regulates the RpoN–RpoS regulatory cascade (Burtnick et al., 2007; Ouyang et al., 2008; Blevins et al., 2009; Richards et al., 2015). Additionally, motility is believed to be regulated by both c-di-GMP and the phosphorylation of CheY (Kostick et al., 2011; Novak et al., 2014; Caimano et al., 2016). These reversible PTMs control a significant number of cellular processes without a significant expenditure of energy. Sterba et al. (2008) demonstrated that, in *B. burgdorferi*, no membrane proteins are glycosylated. Furthermore, succinylation is unlikely in *B. burgdorferi* since its genome does not harbor the genes encoding proteins required for the Krebs cycle therefore it is unable to synthesize succinyl-CoA (Fraser et al., 1997; Gherardini et al., 2010). However, Yang et al. (2018) recently described a method to identify acetylated proteins in *B. burgdorferi* suggesting that this type of PTM was occurring in the Lyme disease spirochete. By using reversible lysine acetylation, *B. burgdorferi* could efficiently maintain control of protein function and cellular physiology without expending energy on transcription and translation to modulate these activities.

Little is known about the effects of this particular PTM of proteins in *B. burgdorferi.* Based upon patterns of lysine acetylation in other bacteria, we hypothesized that acetylation is used by *B. burgdorferi* to modulate the enzymatic activity of proteins required for central metabolism, transcription and translation. This strategy would allow these spirochetes to control energy flux via reversible PTM of proteins in an energy efficient manner. To test this concept, we determined a growth-phase related acetylome in *B. burgdorferi* by using a high sensitivity mass spectrometry-based proteomic approach. Our data suggested that central metabolism and genetic information processing were the two main cellular processes affected by acetylation. We also demonstrated that acetylation directly modulated the activity of glycolytic enzymes using specific enzymatic assays. Additionally, using specific mutant strains deficient in acetate anabolism [B31-A3 Δ*ackA* (Ac-P minus, acetyl-CoA minus) and B31-A3 Δ*pta* (Ac-P plus, acetyl-CoA minus)], we were able to demonstrate that, unlike lysine acetylation in other bacteria, Ac-P accounts for 99% of the acetylation observed in *B. burgdorferi.*

## Results

### The Growth Phase Dependent Acetylome of *B. burgdorferi*

**FIGURE 1:**
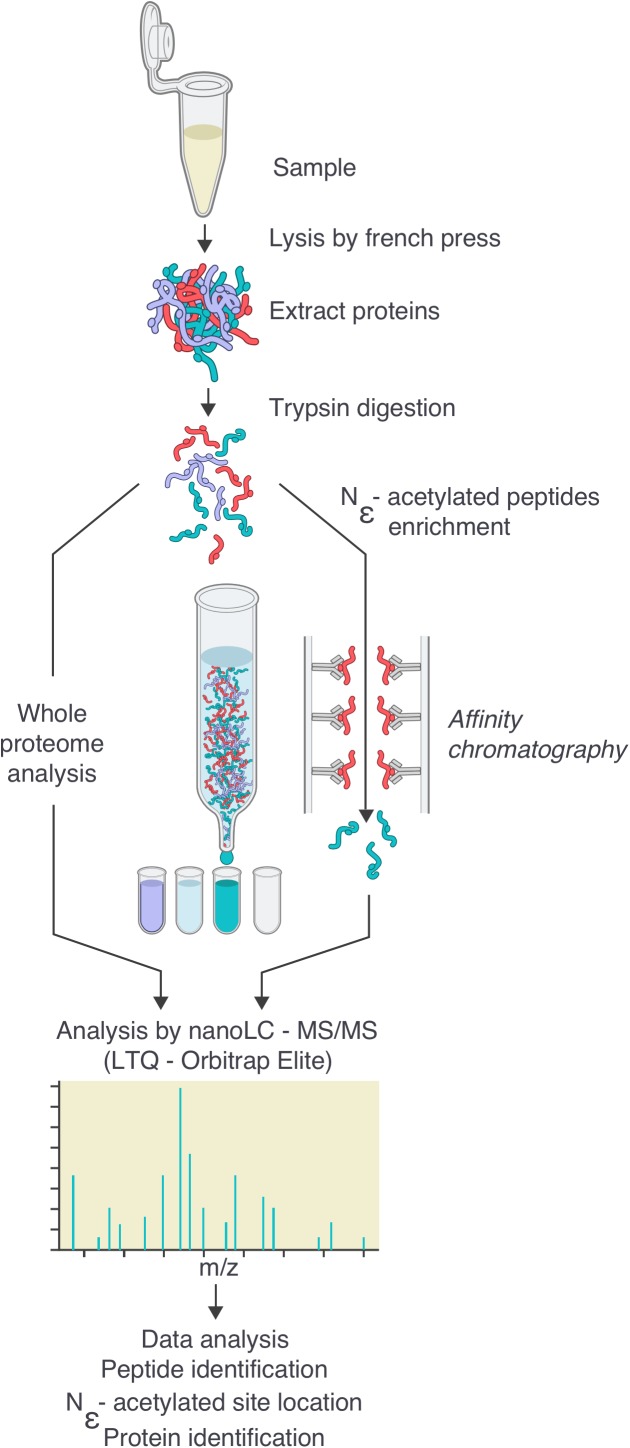
Workflow for the generation of the *Borrelia burgdorferi* acetylome. Cells were grown to the desired density, then washed twice in HN buffer supplemented with deacetylase inhibitor and resuspended in the same buffer. Cells were lysed by French Press. After centrifugation to eliminate cellular debris, proteins were digested by trypsin. Digested peptides were analyzed by nanoLC-MS/MS either directly or after an enrichment by affinity chromatography using pan-acetyllysine antibodies. Data were analyzed by bio-informatics (see the section “Materials and Methods”): peptide and protein identification, acetylated site location, subcellular localization, molecular function.

**FIGURE 2:**
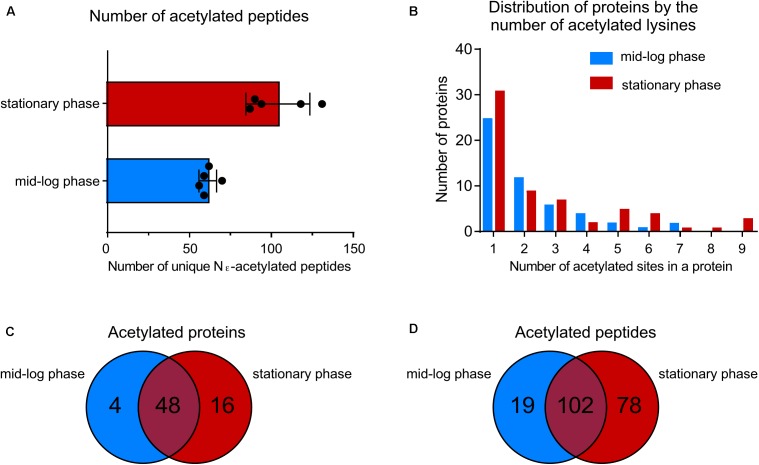
Growth-phase-related acetylation. **(A)** Number of unique acetylated peptides in wild-type strain at ML phase versus S phase. **(B)** Distribution of proteins acetylated by the number of acetylated lysine residues. **(C)** Venn diagram showing the number of acetylated proteins at both growth phases. **(D)** Venn diagram showing the number of unique acetylated peptides at both growth phases.

By using a sensitive mass spectrometry-based proteomics approach, we established the first acetylome of *B. burgdorferi* during mid-log (ML) and stationary (S) phases of growth. Spirochetes were grown to ML and S phase, the cells were lysed and harvested for analyses. Proteins were digested by trypsin and the peptides were analyzed by nanoLC-MS/MS on a high resolution and high accuracy mass spectrometer either directly or after affinity chromatography using pan-anti-acetyllysine antibodies (**Figure 1**). From 5 independent experiments, we identified 61 ± 5 unique acetylated peptides from ML phase cells that originated from 52 distinct proteins and 104 ± 19 unique acetylated peptides from S phase cells that originated from 64 distinct proteins (**Figure 2A** and **Supplementary Table 1**). The number of acetylated sites per protein varied from 1 to 7 in ML phase and up to 9 in S phase (**Figure 2B**). Among the 52 and 64 proteins identified to be acetylated, 4 were uniquely acetylated at ML phase (NusA, RpsG, SpoVG, and BB0367) and 16 uniquely at S phase (NagB, AtpA, GuaB, GlyA, Crr, MurA, ValS, PrfA, RpmC, RplQ, PheT, GlyQS, RplJ, CysS, DnaN, and BB0379) (**Figure 2C** and **Supplementary Table 1**). As shown by the Venn diagrams (**Figures 2C,D**), if a similar set of proteins were acetylated in both phases, these proteins were acetylated at multiple sites at S phase.

**FIGURE 3:**
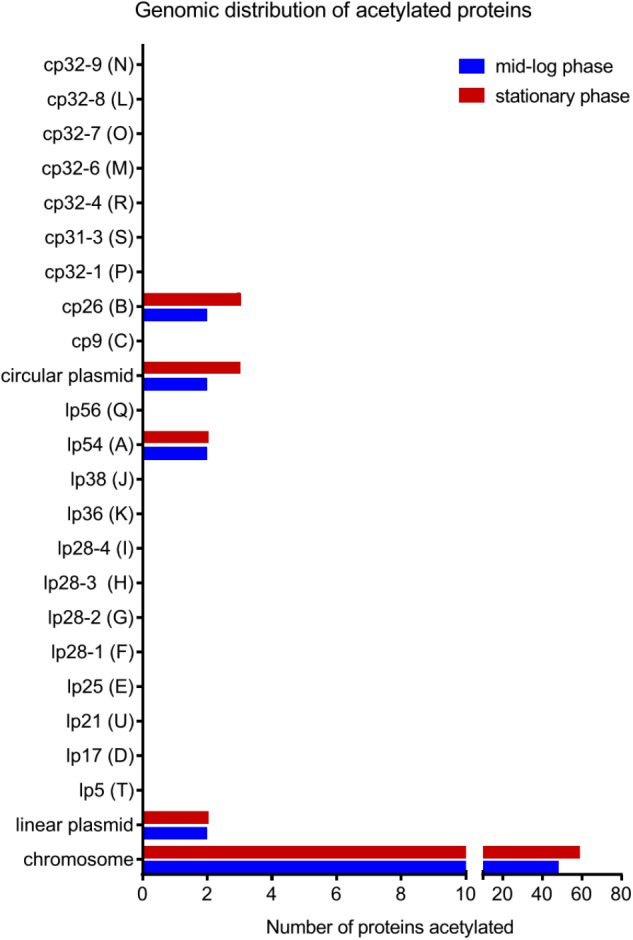
Genomic distribution of acetylated proteins at both growth phases in the wild-type strain.

To further characterize the acetylome, we looked at the localization in the genome of the genes encoding acetylated proteins. *B. burgdorferi* strain B31 has a unique genome composed of a linear chromosome (852 protein-coding ORFs), 9 circular plasmids (311 protein-coding ORFs) and 12 linear plasmids (282 protein-coding ORFs) (Fraser et al., 1997; Casjens et al., 2000). The average number of lysine residues per protein is about 10% regardless of their localization on the genome (**Supplementary Table 2**). Surprisingly, acetylated proteins were encoded by genes primarily found on the linear chromosome (86% and 92% in ML and S phase, respectively) (**Figure 3**). A small set of acetylated proteins localized to plasmids cp26 and lp54. These data suggest an acetylation bias for replicons that harbor genes encoding proteins that are essential for basic, essential physiological functions.

### Predicted Subcellular Localization, Functional Annotation, and Protein Interaction Network of the Acetylated Proteins

**FIGURE 4:**
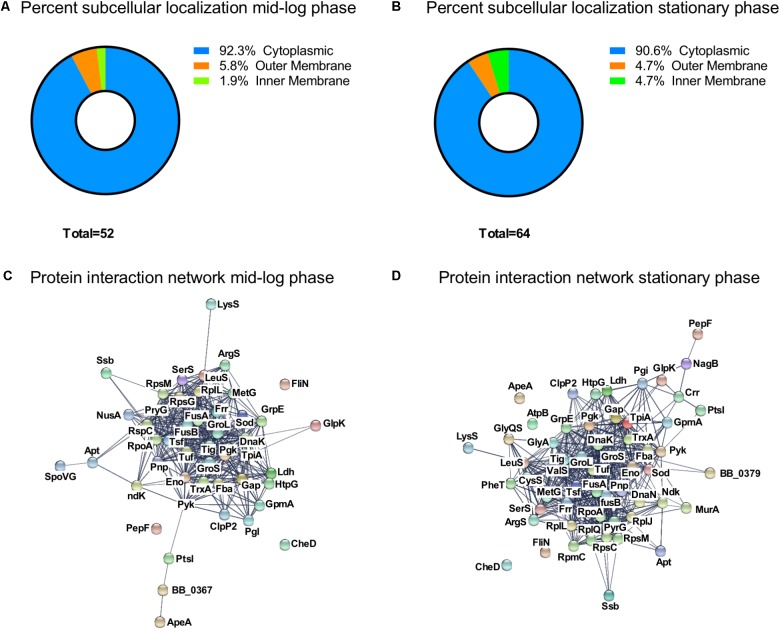
Characterization of the growth-phase-related acetylome. Percent subcellular localization of acetylated proteins at **(A)** ML phase and at **(B)** S phase. Protein interaction network generated by STRING database at **(C)** ML phase and at **(D)** S phase.

We analyzed the subcellular localization of the identified acetylated proteins (**Figures 4A,B**). About 90% of the proteins were localized to the cytoplasm. In ML phase, three proteins were localized to the outer membrane (OspA, OspB, and OspC) and one protein was localized to the inner membrane (AtpA) (**Figure 4A**). In S phase, three proteins were localized to the outer membrane (OspA, OspB, and OspC) and three to the inner membrane (AtpA, AtpB, and Crr) (**Figure 4B**).

To gain further insight into the role of acetylation in cellular processes, we classified the acetylated proteins by function based on the KEGG (Kanehisa and Goto, 2000; Tanabe and Kanehisa, 2012), ERGO (Overbeek et al., 2003), and UniProt (The UniProt Consortium, 2017) databases (**Table 1**). The acetylated proteins were involved in genetic information (18 proteins at ML vs. 25 at S), metabolism and transport (16 proteins at ML vs. 21 at S), protein folding and degradation (eight proteins regardless the growth phase), detoxification (ClpP2, TrxA, and SodA), outer surface protein (OspA, OspB, and OspC), motility and chemotaxis (FliN, CheD). The overall trend in Gene Ontology (GO) distribution of the acetylated proteins was similar to what has been observed in other bacteria.

**Table 1:** Functional classification of the acetylated proteins compared to non-acetylated proteins per GO biological processes.

		Acetylated protein at mid-log phase		Acetylated protein at stationary phase	
	Total # proteins	# Protein	%	# Protein	%
Metabolism	72	15	20.8	19	26.4
Transport	57	1	1.8	2	3.5
Cell wall	20	0	0	1	5.0
Secretion	12	0	0	0	0
Genetic information	43	18	41.9	25	58.1
Protein folding and degradation	15	8	53.3	8	53.3
Detoxification	10	3	30	3	30
Outer surface protein	41	3	7.3	3	7.3
Regulator	14	0	0	0	0
Motility and chemotaxis	53	2	3.8	2	3.8
Cell division	8	0	20.8	0	0
Unknown function	469	2	0.4	1	0.2

To understand the connection between all acetylated proteins, we constructed a protein interaction network map by using the STRING database (Szklarczyk et al., 2015) (**Figures 4C,D**) with a confident score of 0.7. This interaction map showed that 47 of the 52 acetylated proteins found at ML phase were network nodes and connected by 381 direct physical interactions (**Figure 4C**) while 57 of the 64 acetylated proteins found at S phase were network nodes and connected by 502 direct physical interactions (**Figure 4D**). Taken together, these findings demonstrated a strong correlation between identified acetylated proteins and their potential functions.

### Central Metabolism Is Affected by Lysine Acetylation

**FIGURE 5:**
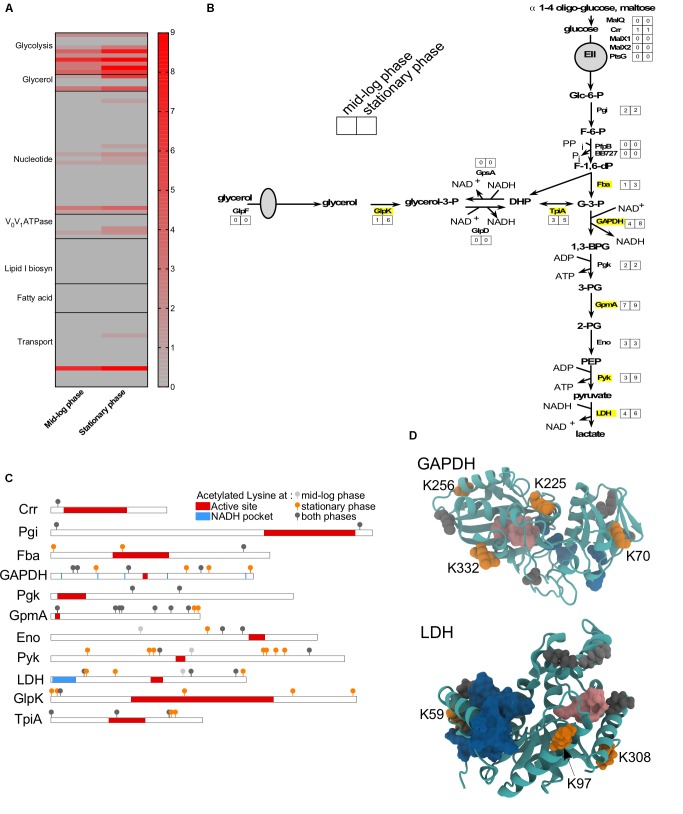
Metabolism is affected by acetylation. **(A)** Heat-map showing the number of acetylated lysines at ML versus S phase in wild-type for all proteins involved in metabolism or transport. **(B)** Glycolysis and glycerol pathways with the number of sites acetylated at each growth phase indicated. Proteins that were more acetylated at S phase than ML phase were indicated in yellow. **(C)** Domain schematics of selected proteins and localization of acetylated lysines. **(D)** 3D structure of GAPDH and LDH proteins showing the potential impact of adding an acetyl group to a lysine residue. **(C,D)** Domains (active site, in red/pink; NAD or NADH pocket, in blue) were based on UniProt and Prosite database. Acetylated lysine were indicated in light gray (only at ML phase), in dark gray (at both growth phase), in orange (only at S phase).

As observed in other bacteria, proteins involved in metabolism and transport were often acetylated (**Figure 5**). Among all the observed proteins, glycolytic enzymes were the mostly commonly targeted (**Figure 5A**). Adaptation to S phase led to increased number of lysine residues acetylated on six glycolytic enzymes (Fba, GAPDH, GmpA, Pyk, LDH, and TpiA) and one enzyme involved in the glycerol metabolism (GlpK) (**Figures 5A,B**). Nakayasu et al. (2017) demonstrated that lysine acetylation on proteins involved in glycolysis is well conserved among bacteria and down-regulates activity of specific enzymes. To determine whether the acetylation of specific lysine residues could modulate protein function, we mapped the different sites of acetylation to known functional domains (**Figures 5C,D**). While the primary sequence of specific proteins did not suggest a potential effect of acetylation on protein function (**Figure 5C**), the tertiary structure revealed that some of the lysine residues acetylated during S phase could potentially affect enzyme activity (**Figure 5D**). In several cases, acetylated lysine residues were localized in a way that could obstruct or directly impact the function of the active site. For example, the GAPDH active site could be affected by acetylation of lysine K225 and/or K332 (**Figure 5D**). Likewise, LDH activity could be affected by acetylation of lysine K59 in the active site of this enzyme (**Figure 5D**). Our data suggests that acetylation could possibly modulate these enzyme activities.

### BdrQ Was Not Required for Acetylation

BdrQ (BBN34) is annotated as a member of the *Borrelia* direct repeat family by Fraser et al. (1997) and contains the 20 conserved amino acids (**Supplementary Figure 1A**) found in the 13 other members of the BDR-repeat family proteins. Recently, Hentchel and Escalante-Semerena (2015) proposed that BdrQ is an acetyl-CoA dependent Gcn5-related *N*-acetyltransferase (GNAT). To test this hypothesis, we assayed a strain missing this gene. Because *bdrQ* localizes to cp32-9, which harbors a large number of repeat sequences adjacent to this locus, we were unable to inactivate this gene by targeted mutagenesis. Therefore, we used strains B31-C1 (wild-type strain missing cp32-9) and B31-C1 complemented with *bdrQ* on shuttle vector pBSV2 (B31-C1 pBSV2::P*_flgB_*-*bdrQ*) to determine the role of BdrQ on protein acetylation. We grew these strains to S phase and analyzed cell lysates by immunoblotting to determine if there was a difference in the acetylation profile of these two strains (**Supplementary Figure 1B**). No difference was observed between the two strains. Our data suggested that BdrQ was not involved in acetylation.

### Acetyl-Phosphate Is the Primary Source of Acetylation

**FIGURE 6:**
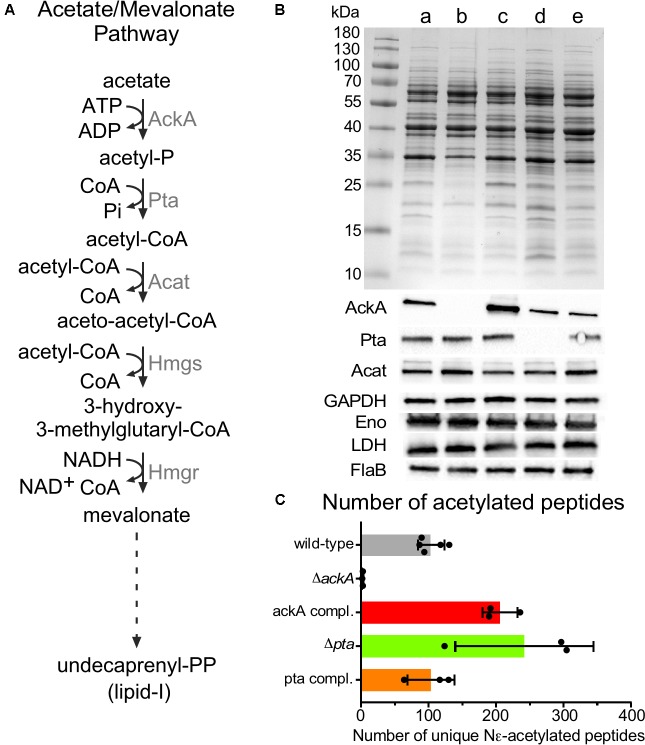
Acetylome in the Δ*ackA*, Δ*pta* and their complemented strains. **(A)** Acetate/Mevalonate pathway in *B. burgdorferi*. **(B)** Wild-type (lane a), Δ*ackA* (lane b), Δ*pta* (lane d), *ackA* complemented (lane c), and *pta* complemented (lane e) strains were grown in BSK-II with 500 μM of mevalonolactone. Cell lysates were analyzed by SDS-PAGE, coomassie blue staining and immunoblotting by probing with AckA, Pta, Acat, GAPDH, Eno, LDH, and FlaB antigen-specific antisera. Uncropped gels are displayed in **Supplementary Figure 4**. **(C)** Number of unique acetylated peptides in wild-type, *ackA*, Δ*pta* and their complemented strains.

With the growth-phase-related acetylome of *B. burgdorferi* established, we then sought to determine the acetyl donor. Compared to other bacteria, where the production of acetyl-CoA and Ac-P are derived from multiple metabolic pathways, *B. burgdorferi* is unique in that its acetate metabolism is limited to a single metabolic pathway which synthesizes lipid I for cell wall biogenesis (mevalonate pathway) (Richards et al., 2015) (**Figure 6A**). In this pathway, acetate is converted to Ac-P by acetate kinase (AckA) and to acetyl-CoA by phosphotransacetylase (Pta). Three acetyl-CoA moieties are condensed to form mevalonate which in turn is converted to undecaprenyl phosphate further and to lipid I (Van Laar et al., 2012; Richards et al., 2015). To define this pathway, Richards et al. (2015) generated B31-A3-Δ*ackA*, B31-A3-Δ*pta* and their corresponding complements. Analysis by immunoblotting, demonstrated that AckA and Pta were not produced in their respective mutants. In the *ackA* complemented strain, AckA was expressed at a higher level than wild-type. However, it was not surprising since *ackA* was reintroduced into the mutant strain on a multi-copy plasmid pBSV2 (Richards et al., 2015). Surprisingly, in the *pta* complemented strain, Pta was not overexpressed even though the complementation was also done on a multi-copy plasmid. Inactivation of *ackA* or *pta* did not affect metabolism (Acat, GAPDH, Eno, and LDH) or motility (FlaB) (**Figure 6B**) when supplemented with mevalonate.

The acetylome of S phase cells was determined using B31-A3-Δ*ackA* (Ac-P^-^, acetyl-CoA^-^), B31-A3-Δ*pta* (Ac-P^+^, acetyl-CoA^-^) and their respective complements to determine whether AcP or acetyl-CoA was the main donor for lysine acetylation (**Figure 6C**). The Δ*ackA* mutant showed nearly no acetylation (2.6 ± 0.6 unique peptides) which matched with only two distinct proteins (**Figure 6C** and **Supplementary Table 1**) while the wild-type strain produced 104 ± 19 unique peptides that localized to 64 proteins, (**Figures 2A**, **6C** and **Supplementary Table 1**). A dramatic increase in acetylation was observed in the *ackA* complemented strain (206 ± 26 unique peptides) which matched with 115 distinct proteins, (**Figure 6C** and **Supplementary Table 1**). Hyper-lysine acetylation was observed in the Δ*pta* mutant (242 ± 102 unique peptides which matched with 144 distinct proteins) (**Figure 6C** and **Supplementary Table 1**) while the Δ*pta* complemented strain displayed a similar level of acetylation (103 ± 35 unique peptides and 78 proteins, **Figure 6C** and **Supplementary Table 1**) as the wild-type (104 ± 19 unique peptides, **Figure 6C** and **Supplementary Table 1**). We believe that the hyper-acetylation observed in the Δ*pta* mutant is the result of the accumulation of Ac-P which cannot be converted to acetyl-CoA in the absence of Pta. Interestingly, in the Δ*ackA* complemented strain, the Δ*pta* mutant and the *pta* complemented strains, proteins in the mevalonate pathway were acetylated [AckA and HmgS in the *ackA* complemented strain, Acat in the Δ*pta* mutant and AckA and Acat in the *pta* complemented strain (**Supplementary Table 1**)]. This observation suggests that the cell may directly down-regulate the pathway when Ac-P and/or acetyl-coA are over-produced to conserve ATP. These analyses strongly suggest that Ac-P was the primary source of acetylation in *B. burgdorferi*.

### Lysine Acetylation Directly Affected *in vivo* and *in vitro* Enzyme Activities

**FIGURE 7:**
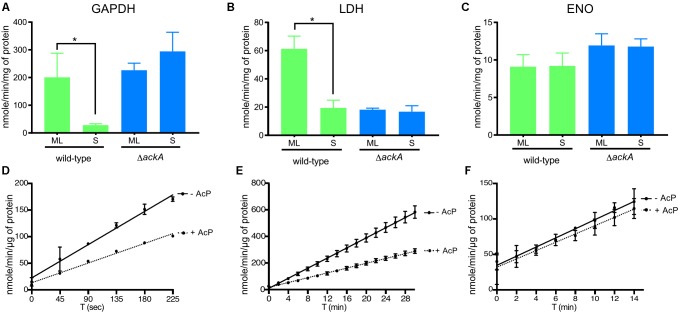
Lysine acetylation directly affected the enzymatic activities of proteins involved in glycolysis. Enzyme activities of GAPDH **(A)**, LDH **(B)**, and Eno **(C)** were measured from crude extracts of the wild-type and the Δ*ackA* mutant at ML and S phase and normalized with total protein (see the section “Materials and Methods”). Effect of acetylation by Ac-P on enzymatic activity assayed in the presence or absence of Ac-P on GAPDH **(D)**, LDH **(E)** or Eno **(F)** purified proteins. For LDH and GAPDH, one unit is the amount of enzyme able to generate 1 μmole of NADH per minute (nmole/min/μL). For Eno, one milliunit is the amount of enzyme able to generate 1 nmole of H_2_O_2_ per minute (nmole/min/μL). Results are expressed at nmole/min/mg of total protein or μg of purified protein. Results reported are the average of at least three independent experiments. Data represent means ± standard deviation. An asterisk indicates a significant difference using a one-way ANOVA where *p* < 0.05.

Finally, to gain insight into the role of lysine acetylation on enzyme function, we measured the enzyme activity of glyceraldehyde 3-phosphate dehydrogenase (GAPDH) and L-lactate dehydrogenase (LDH) at ML and S phase to determine whether the increased acetylation observed in S phase affected enzyme activity (**Figure 7**). We previously showed that four lysine residues within GAPDH and four lysine residues within LDH (**Supplementary Table 1**) were acetylated in ML phase cells while both proteins were more robustly acetylated (six sites within LDH and eight within GAPDH) during S phase. Therefore, we measured GAPDH and LDH specific activities in cell lysates isolated from wild-type and Δ*ackA* mutant cells (Ac-P^-^, Acetyl-CoA^-^; no detectable lysine acetylation of metabolic enzymes) (**Figures 7A,B**). The enzymatic activities of GAPDH and LDH decreased by 85% and 68%, respectively, between ML and S phase in the wild-type strain (**Figures 7A,B**). In the Δ*ackA* mutant cell lysates, the specific activities of GAPDH were similar in both ML and S phase to the enzyme activities measured in ML phase of the wild-type B31 cell lysates (**Figure 7A**). These data suggested that robust lysine acetylation inhibited GAPDH activity. In contrast, the specific activity of LDH was low in the Δ*ackA* mutant cell lysates, at levels comparable to the those observed in wild-type cells in S phase (**Figure 7B**). These data suggested that some intermediate level of acetylation of LDH was required for maximum activity while hyper- or non-acetylation of LDH yielded reduced enzyme activity. As an internal control, we also analyzed enolase (Eno) activity in cell lysates since this enzyme has the same lysine acetylation pattern (two acetylated lysine residues) regardless of the growth phase (**Figure 7C**). Enolase activity remained at the same level regardless the growth phase. We also confirmed by immunoblot that similar amounts of protein were produced regardless of genetic background or growth phase (**Supplementary Figure 2**).

To confirm that acetylation can affect the enzyme activity of LDH and GAPDH, we determined the specific activities of purified enzymes after incubation with Ac-P (**Figures 7D,E** and **Supplementary Figure 3**). Acetylation decreased the activity of GAPDH by 41% and LDH by 52% while Eno activity was not affected by acetylation (**Figure 7F**). Incubating purified LDH with Ac-P did not result in an increase in enzymatic activity that mimicked the increase in activity observed in cell lysates from ML phase cells (**Figures 7B,E**). This suggested that *in vitro* acetylation of LDH resulted in hyper-acetylation of the enzyme that inhibited activity. Since this enzyme generates most of the reducing equivalents (NAD^+^) for central metabolism, its activity is central for the metabolic well-being of *B. burgdorferi.* More enzyme studies on LDH are needed to understand this important enzyme and its PTMs. Taken together, these data indicated that Ac-P was the primary acetyl donor in *B. burgdorferi* and lysine acetylation could modulate protein function in the Lyme spirochete.

## Discussion

Over the last 5 years, numerous studies show the relevance of lysine acetylation in bacteria (Hu et al., 2010; Bernal et al., 2014; Cain et al., 2014; Hentchel and Escalante-Semerena, 2015; Ouidir et al., 2016; Wolfe, 2016; Carabetta and Cristea, 2017; Ren et al., 2017). This PTM plays an important role in several cellular processes such as central metabolism, transcription, and translation (Bernal et al., 2014; Cain et al., 2014; Carabetta and Cristea, 2017). Lysine acetylation affects the enzymatic activity of target proteins (Starai et al., 2005; Gardner et al., 2006; Gardner and Escalante-Semerena, 2008; Thao and Escalante-Semerena, 2011; Crosby et al., 2012; Hayden et al., 2013; Tucker and Escalante-Semerena, 2013; Vergnolle et al., 2016; Venkat et al., 2017), or modulates the binding of transcriptional regulators to their target sequences (Hu et al., 2013; Castano-Cerezo et al., 2014; Amin et al., 2016; Ghosh et al., 2016; Sang et al., 2016). In bacteria, acetylation occurs via enzymatic and/or non-enzymatic mechanisms (Hentchel and Escalante-Semerena, 2015; Tu et al., 2015; Carabetta and Cristea, 2017). During enzymatic acetylation, a lysine acetyltransferase (KAT, Gcn5 *N*-acetyltransferase superfamily in bacteria) uses an acetyl-CoA to transfer the acetyl moiety to the target lysine residue (Hentchel and Escalante-Semerena, 2015). In all cases, these reactions are dependent on specific amino acid sequences adjacent to the target lysine residue (Carabetta and Cristea, 2017). Lysine acetylation can occur via non-enzymatic reactions, which use acetyl-phosphate or acetyl-CoA as a donor (Wagner and Hirschey, 2014; Carabetta and Cristea, 2017). In these cases, lysine acetylation is not specifically targeted and no amino acid sequence specificity is required. Deacetylation of modified lysine residues requires deacetylases that remove the acetyl-group (Hentchel and Escalante-Semerena, 2015; Tu et al., 2015; Carabetta and Cristea, 2017).

We established the first growth-phase related acetylome in *B. burgdorferi*. As reported in other bacteria, a relatively low number (5%) of the potential encoded proteins of *B. burgdorferi* were acetylated. Proteins were mainly involved in central metabolism and genetic information processes (transcription, translation, etc.) (**Figures 2**, **3** and **Supplementary Table 1**). Interestingly, these critical cell functions were targeted during both ML and S phases of growth. However, acetylation of target proteins in ML phase was limited to a few lysine residues while these same proteins were acetylated at multiple sites during S phase. No major regulator was found to be acetylated during either stage of growth. It is important to note that our analyses of lysine acetylation in ML phase does not reflect the overall acetylation of target proteins within the cell population. For example, it is difficult to assess the percentage of a particular proteins acetylation in a given population. This is the result of the sensitivity of the methods used and the heterogeneity of the cells within this population.

Importantly, analyses of B31-A3-Δ*ackA* and B31-A3-Δ*pta* mutant strains demonstrated that Ac-P is the primary source of acetylation and suggest that lysine acetylation in *B. burgdorferi* is a non-enzymatic process (**Figure 6**). However, similar sets of proteins were observed in other bacteria (Meng et al., 2016) to be acetylated, meaning that if the process is not enzymatic, it cannot be completely random. Nakayasu et al. (2017) showed that lysine acetylation is a conserved mechanism that controls glycolytic enzymes. Here, we observed that the enzyme activities of GAPDH and LDH are down-regulated by acetylation, while enolase was not (**Figure 7**). Our observations, associated with previous studies (Hu et al., 2010; Hentchel and Escalante-Semerena, 2015; Wolfe, 2016; Carabetta and Cristea, 2017; Nakayasu et al., 2017), strengthen the idea that acetylation is used to control metabolism. In *B. burgdorferi*, which has limited metabolic capacity (Fraser et al., 1997; Gherardini et al., 2010), the two key enzymes (GAPDH and LDH) that insure the appropriate NAD^+^/NADH ratio, a marker of the cell vitality, are affected. Using specific enzymatic assays, we were able to demonstrate that hyper-acetylation of LDH in S phase appeared to decrease the enzymatic activity of this enzyme. Surprisingly, the lack of acetylation also negatively affected the activity of this protein (**Figure 7B**), which provides a new insight into understanding this PTM. Acetylation seems to be a complex process that can both up and/or down-regulate the enzymatic activity of a target protein. More studies are necessary to better understand how the acetylation of key lysine residues of enzymes involved in central metabolism correlates with *B. burgdorferi* fitness and long-term survival.

Previous studies have demonstrated that protein acetylation affects the enzymatic activity and stability of target proteins in numerous bacteria (Starai et al., 2005; Gardner et al., 2006; Gardner and Escalante-Semerena, 2008; Thao and Escalante-Semerena, 2011; Crosby et al., 2012; Hayden et al., 2013; Tucker and Escalante-Semerena, 2013; Sang et al., 2016; Vergnolle et al., 2016; Venkat et al., 2017). This also seems to be the case in *B. burgdorferi.* Since proteins involved in translation were highly acetylated (e.g., ribosomal proteins, aminoacyl-tRNA ligase, etc.), it seems reasonable to speculate that protein synthesis is similarly affected in *B. burgdorferi*. In addition, various chaperones and proteases were also acetylated which could affect protein turnover by increasing the half-life and stability of target proteins. This mechanism could conserve energy and enhance long-term survival in a limited nutrient environment. Furthermore, proteins involved in metabolism, particularly those in glycolytic pathways, were acetylated which could potentially protect these enzymes while controlling their enzymatic activity. Currently, we hypothesize that lysine acetylation is used to modulate enzyme activity and long-term stability of proteins during the long-term survival of the bacteria in the tick midgut between blood meals. This strategy would allow the bacteria to activate key glycolytic enzymes by deacetylation rather than expending energy to synthesize new proteins via new transcription and translation of the genes encoding key enzymes. This would be an appealing, low-energy strategy for a bacterium with limited metabolic capabilities. Future work will be focused on: (i) characterizing how the acetylation of lysine residues in key glycolytic enzymes are impacted by acetylation, (ii) identifying and characterizing the key deacetylase(s) that are involved in reversing lysine acetylation, and iii) assessing the impact of acetylation on the *in vivo*, long-term survival of the B31-A3-Δ*ackA* and B31-A3-Δ*pta* mutants in ticks. The data presented here and future studies of the role of lysine acetylation in *B. burgdorferi*, will provide valuable insights into the role of this PTM on the pathogenesis of this important spirochete.

## Materials and Methods

### Bacterial Strains, Media, and Growth Conditions

The bacteria strains used in the study are wild-type B31-A3 and B31-C1 (Elias et al., 2002), B31-A3-Δ*ackA*, B31-A3-Δ*pta*, B31-A3-Δ*ackA* complement (Δ*ackA*::pCR200) and B31-A3 *pta* complement (Δ*pta*::pCR201) (Richards et al., 2015). *Borrelia* strains were grown in BSK-II media (Barbour, 1984), pH 6.8, 450 mOsM at 34°C under an atmosphere of 3% O_2_, 5% CO_2_ balance N_2_. For strains B31-A3-Δ*ackA*, B31-A3-Δ*pta*, 500 μM of mevalonolactone (M4667, Millipore Sigma, St. Louis, MO, United States) was added to the growth media. Antibiotics were used at the following concentrations: gentamicin, 20 μg/mL (G1264, Millipore Sigma, St. Louis, MO, United States) or streptomycin, 50 μg/mL (S9137, Millipore Sigma, St. Louis, MO, United States). Cell densities were determined by dark-field microscopy.

*Escherichia coli* strains were grown in LB (240230, BD, Franklin Lakes, NJ, United States) (Bertani, 2004). Antibiotics were used at the following concentrations: kanamycin at 50 μg/mL (1355006, Millipore Sigma, St. Louis, MO, United States) or ampicillin at 100 μg/mL (A1593, Millipore Sigma, St. Louis, MO, United States).

Shuttle vector, pBSV2 (ATCC 87806), containing P_flgB_-*bdrQ* was synthesized by GenScript, Piscataway, NJ, United States. B31-C1 cells were transformed with pBSV2::P_flgB_-*bdrQ* as described previously by Samuels (1995).

### Preparation of Protein Samples

Cells were harvested at ML phase (8 × 10^6^ cells/mL) or at stationary phase (48 h after reaching 10^8^ cells/mL). Cells were washed twice in HN buffer (50 mM HEPES pH 7.5, 50 mM NaCl) supplemented with 0.3 μM trichostatin A (T8552, Millipore Sigma, St. Louis, MO, United States) and 20 mM nicotinamide (72340, Millipore Sigma, St. Louis, MO, United States) as deacetylase inhibitors. The cell pellets were resuspended in the same buffer and disrupted by three passages through a pre-cooled French pressure cell at 12,000 psi. Cell debris was removed by centrifugation (7,000 × g, 30 min, 4°C) And cell lysates were aliquoted and stored at -80°C. Protein concentration was determined with the Pierce^TM^ BCA Protein Assay kit (23225, Thermo Scientific, Rockford, IL, United States) using BSA as a standard.

### Enrichment of Acetylated Lysine Containing Peptides

Proteins (3 mg) were solubilized in 6 M urea and 15 mM DTT for 1 h. The reduced proteins were alkylated with 15 mM iodoacetamide for 45 min. Proteins were precipitated with ice-cold acetone (-20°C, 2 h). The protein pellet was suspended in ammonium bicarbonate (pH 8, 10 mM) and digested with trypsin (enzyme/protein ratio: 1/50) overnight at 37°C with shaking. Peptides were dried and stored at -20°C.

Peptide enrichments were performed following the manufacturer’s instruction (PTM Biolabs). Briefly, 60 μL of anti-acetyllysine polyclonal antibodies conjugated to protein A-Sepharose and washed three times with the IP buffer (100 mM NaCl, 1 mM EDTA, 50 mM Tris-base pH 8). Tryptic digests were suspended in the IP buffer, mixed with the conjugated beads, and incubated at 4°C overnight with gentle shaking. Beads were washed three times with IP buffer and three times with water. Peptides were eluted from the beads by three washes with formic acid (FA) 1%. After enrichment, peptides were then desalted on a C_18_ tip column, dried with a speed vac and stored at -20°C until MS analysis.

### Mass Spectrometry Analyses of Lysine Acetylated Peptides

All experiments were performed on a LTQ-Orbitrap Elite coupled to an Easy nLC II system (both from Thermo Scientific). Samples were injected onto an enrichment column (C_18_ PepMap100, Thermo Scientific) and separation was achieved with an analytical column needle (NTCC-360/100-5-153, Nikkyo Technos). The mobile phase consisted of H_2_O/FA 0.1% (buffer A) and ACN/FA 0.1% (buffer B). Tryptic peptides were eluted at a flow rate of 300 nL/min, using a three-step linear gradient: from 2 to 40% B over 75 min, from 40 to 80% B in 4 min and at 80% B for 11 min. The mass spectrometer was operated in positive ionization mode with capillary voltage and source temperature set at 1.5 kV and 275°C, respectively. The samples were analyzed using CID (collision induced dissociation) method. The first scan (MS spectra) was recorded in the Orbitrap analyzer (*R* = 60,000) with the mass range m/z 400–1800. Then, the 20 most intense ions were selected for MS2 experiments. Singly charged precursors were not selected for a MS2 analysis. Dynamic exclusion of already fragmented precursor ions was applied for 30 s, with a repeat count of 1, a repeat duration of 30 s and an exclusion mass width of ±5 ppm. The precursor isolation width was 2 *m/z*. Fragmentation occurred in the linear ion trap analyzer with normalized collision energy of 35. All measurements in the Orbitrap analyzer were performed with on-the-fly internal recalibration (lock mass) at m/z 445.12002 (polydimethyl cyclosiloxane).

### Database Search

Raw data files were processed using Proteome Discoverer 1.4 software (Thermo Scientific). Peak lists were searched using the MASCOT search software (Matrix Science) against the database *B. burgdorferi*. Database searches were performed with the following parameters: two missed trypsin cleavage sites allowed; variable modifications: carbamidomethylation on cysteine, oxidation on methionine, and lysine acetylation. The parent-ion and daughter-ion tolerances were 5 ppm and 0.35 Da, respectively. False discovery rate (FDR) threshold for identifications was specified at 1% (for proteins and peptides). For each identification, we looked carefully at the ion score (above 19), the peptide rank, the daughter ions (b- and y-fragment ion series), and the expectation value (E-value, ≤0.05). We checked manually all spectra containing acetylation to ensure both the location of the PTM and the peptide sequence (**Supplementary Figure 5**).

### Bioinformatics Annotation of Lysine-Acetylated Peptides

All identified acetylated peptides were compared to the UniProt database (The UniProt Consortium, 2017) using *B. burgdorferi* (strain ATCC 35210/B31/CIP 102532/DSM 4680), taxon identifier 224326 as reference. Proteins were classified by GO based on biological process, subcellular localization and molecular function using the UniProt database (The UniProt Consortium, 2017) and ERGO (Overbeek et al., 2003). The Kyoto Encyclopedia of Genes and Genomes (KEGG) (Kanehisa and Goto, 2000; Tanabe and Kanehisa, 2012) was used to annotate the metabolic pathways. A protein interaction network was generated by using the STRING database (Szklarczyk et al., 2015). All networks were made with a confidence score in the interactions of 0.7 (high confidence). The Venn diagrams were generated by using the Venn diagram^1^ from the University of Ghent, Belgium.

### Enzyme Modeling

Domains (i.e., active site) on proteins were defined by using the Prosite database (Sigrist et al., 2002, 2005, 2013; Hulo et al., 2008). The structures of LDH and GAPDH were predicted using I-TASSER (Yang et al., 2015). The correctness of the modeled structure was evaluated by a confidence score (*C*-score) (Zhang, 2008). *C*-scores are normally in the range of [-5, 2]. A higher *C*-score indicates more confidence in the model, with a cutoff *C*-score > -1.5 indicating models with a very high likelihood of correct topology (Zhang, 2008). I-TASSER combines available state-of-the art threading programs and ranks the templates by sequence and structure based scores. Top templates from each threading program are then selected for further refinement. Typically, I-TASSER returns five top models with different *C*-scores. However, for LDH and GAPDH, I-TASSER returned only one model indicating the agreement between different threading programs on the output model. A *C*-score ranging between [1.22, 2.00] for these outputs also indicates very high likelihood the models having correct topology. The figures of the models were created by VMD – Visual Molecular Dynamics (Humphrey et al., 1996).

### Enzyme Assays

L-lactate dehydrogenase activity was measured by using the lactate dehydrogenase activity assay kit (MAK066, Sigma-Aldrich, St. Louis, MO, United States). The kit follows the conversion of lactate to pyruvate, which generates NADH that reacts with a developer to form a colored product (450 nm). One unit (nmole/min/mL) is the amount of enzyme able to catalyze the conversion of lactate into pyruvate to generate 1 μmole of NADH per minute.

Enolase activity was measured by using the enolase activity assay kit (MAK178, Sigma-Aldrich, St. Louis, MO, United States). Activity is followed by a coupled enzyme assay in which D-2-phosphoglycerate is converted to phosphoenolpyruvate (PEP), resulting in the production of an intermediate that reacts with a peroxidase subtract, producing a colorimetric product (570 nm). One milliunit (nmole/min/μL) is the amount of enzyme able to generate 1 nmole of H_2_O_2_/min.

Glyceraldehyde-3-phosphate (GAP) dehydrogenase activity was measured by using the Glyceraldehyde-3-Phosphate Dehydrogenase Activity Assay Kit (ab204732, Abcam, Cambridge, MA, United States). The kit follows the conversion of GAP to 1,3-bisphosphate glycerate (BPG), which generates an intermediate that reacts with a developer to form a colored product (450 nm). One unit (nmole/min/μL) is the amount of enzyme able to generate 1 μmole of NADH/min.

Activities were reported as nmol/min/mg of protein. Total amount of protein was used to normalize enzyme activities from crude extract. Protein concentration was determined with the Pierce^TM^ BCA Protein Assay Kit (23225, Thermo Scientific, Rockford, IL, United States) using BSA as a standard.

### Protein Purification

The optimized *B. burgdorferi* genes of L-lactate dehydrogenase (*LDH*/*bb0087*), GAP dehydrogenase (*gapdh*/*bb0057*) and Enolase (*eno*/*bb0337*) were cloned into pET30a (LDH and GAPDH) or pET19b (Eno) by GenScript, Piscataway, NJ, United States. LDH and GAPDH were overexpressed and purified by GenScript. Eno was overexpressed and purified as previously described. Briefly, *E. coli* BL21 (DE3) was transformed with the appropriate vector. Transformants were grown in LB at 37°C for LDH and GAPDH and at 18°C for Eno and induced by adding 1 mmol IPTG. Purification was performed using a nickel affinity column and protein concentrations were determined using a Pierce^TM^ BCA Protein Assay Kit (23225, Thermo Scientific, Rockford, IL, United States) with BSA as a standard.

### *In vitro* Acetylation Assay

Purified LDH (0.057 mg/mL), GAPDH (0.085 mg/mL), or Eno (0.0025 mg/mL) proteins were incubated with 1 mM Ac-P (01409, Millipore Sigma, St. Louis, MO, United States) in PBS buffer pH 7.2. The mixtures were incubated at 37°C for 1 h prior to enzymatic assays (**Figures 7D–F**) and western blot (**Supplementary Figure 3**).

### SDS-PAGE and Immunoblotting

Cell lysates were analyzed by immunoblot using a standard protocol. The protein concentration was determined on a Take3 micro-volume plate in a Synergy 2 Multi-Mode plate reader (BioTek Instruments, Winooski, VT, United States). Equal amounts of protein were separated in a AnyKd pre-cast SDS-PAGE gel (Bio-Rad, Hercules, CA, United States) and transferred to a nitrocellulose membrane or PVDF membrane using a Trans-Blot^®^ Turbo^TM^ blotting system (Bio-Rad, Hercules, CA, United States) with a pre-programmed protocol (2.5 A, up to 25 V, 3 min). Blots were probed with antisera and imaged using Super Signal Pico chemiluminescent substrate kit (Thermo Scientific, Rockford, IL, United States) on a ChemiDoc MP system (Bio-Rad, Hercules, CA, United States).

Rabbit polyclonal antisera directed against AckA, Pta, Acat, LDH, Eno, GAPDH were prepared by GenScript, Piscataway, NJ, United States. For immunoblotting, the following dilutions of primary sera were used: anti-AckA, 1:1,000; anti-Pta, 1:250; anti-Acat, 1:1,000; anti-LDH, 1:1,000; anti-Eno, 1:1,000; anti-GAPDH, 1:1,000; anti-FlaB, 1:1,000 (Barbour et al., 1986) or anti-acetyllysine, 1:2,000 (PTM Biolabs, Chicago, IL, United States). Coomassie Blue straining was used as loading control for polyacrylamide gel electrophoresis and Ponceau S staining was used for detection of proteins following transfer to nitrocellulose or PVDF membranes.

## Data Availability

The mass spectrometry proteomics data have been deposited to the ProteomeXchange Consortium via the PRIDE (Vizcaino et al., 2016) partner repository with the dataset identifier PXD010065.

## Author Contributions

SB-G, RC, JH, and FG designed and supervised the study. SB-G wrote the paper. SB-G, CR, JS, SR, KL, JL, and DD performed the experiments except the mass spectrometry experiments. CG, TK, and JH performed the proteomic analysis (enrichment, mass spectrometry, and PTM and protein identifications). All authors reviewed the manuscript.

## Conflict of Interest Statement

The authors declare that the research was conducted in the absence of any commercial or financial relationships that could be construed as a potential conflict of interest.

## Footnotes

1 http://bioinformatics.psb.ugent.be/webtools/Venn/
