# Supplementary material for: Global Profiling of Lysine Acetylation in Borrelia burgdorferi B31 Reveals Its Role in Central Metabolism
Source: Front Microbiol. 2018 Aug 31;9:2036. doi: 10.3389/fmicb.2018.02036 (PMC6127242; doi:10.3389/fmicb.2018.02036)
Supplement: Supplementary file 3 [file Data_Sheet_1.docx]

Supplementary Material

Global Profiling of Protein Lysine Acetylation in *Borrelia burgdorferi* B31 Reveals Its Role in Central Metabolism

Sébastien Bontemps-Gallo^1#^, Charlotte Gaviard^2,3^, Crystal L. Richards^1^, Takfarinas Kentache^2,3^, Sandra J Raffel^1^, Kevin A. Lawrence^1^, Joseph C. Schindler^4^, Joseph Lovelace^4^, Daniel P. Dulebohn^1^, Robert G. Cluss^4^, Julie Hardouin^2,3^, Frank C. Gherardini^1^*

*** Correspondence:** Frank C. Gherardini [fgherardini@niaid.nih.gov](mailto:fgherardini@niaid.nih.gov)

**Supplementary Figure** 1**.** BdrQ is not involved in acetylation. (A) Schematic of BdrQ. In blue, the 20 amino acids at the C-terminus defined as the Bdr membrane-spanning domain. (B) Immunoblot of B31-C1 and B31-C1 pBSV2::P*_flgB_*-bdrQ probed with anti-acetyllysine sera.

**Supplementary Figure 2.** Effect of growth phase on GAPDH, LDH and Eno synthesis. The wild-type and the Δ*ackA* mutant were grown in BSK-II with 500 μM of mevalonolactone until mid-log (ML) and stationary (S) phase. Cell lysates were analyzed by SDS-PAGE immunoblotting by probing with GAPDH (A), Eno (B) and LDH (C) antigen-specific antisera and Coomassie blue staining (D) as loading control.

**Supplementary Figure 3.** Effect of *in vitro* acetylation on GAPDH (A), LDH (B), and Eno (C) treated with acetyl-phosphate (+). Samples from Figures 7 D-F were analyzed by SDS-PAGE, coomassie blue (upper panels) staining and immunoblotting by probing with anti-acetyllysine sera (lower panels) to show that equal amounts of protein were detected using each detection method.

**Supplementary Figure 4.** Uncropped gel of Figure 6B. Wild-type (lane a), Δ*ackA* (lane b), Δ*pta* (lane d), *ackA* complemented (lane c) and *pta* complemented (lane e) strains were grown in BSK-II with 500 μM of mevalonolactone. Cell lysates were analyzed by SDS-PAGE, coomassie blue staining and immunoblotting by probing with AckA, Pta, Acat, GAPDH, Eno, LDH, and FlaB antigen-specific antisera.

**Supplementary Figure 5.** Examples of mass spectra of peptides identified using mass spectrometry.

**Supplementary** **Table 1**. List of proteins acetylated at mid-log and stationary phase in B31-A3 wild-type strain and at stationary for B31-A3 Δ*ackA*, B31-A3 Δ*pta*, B31-A3 Δ*ackA*::pCR200 and B31-A3 Δ*pta*::pCR201 (Richards et al., 2015).

**Supplementary** **Table 2.** List of all proteins with their number of amino acids, lysine residues.
