# Supplementary figures and images for: Global Profiling of Lysine Acetylation in Borrelia burgdorferi B31 Reveals Its Role in Central Metabolism
Source: Front Microbiol. 2018 Aug 31;9:2036. doi: 10.3389/fmicb.2018.02036 (PMC6127242; doi:10.3389/fmicb.2018.02036)

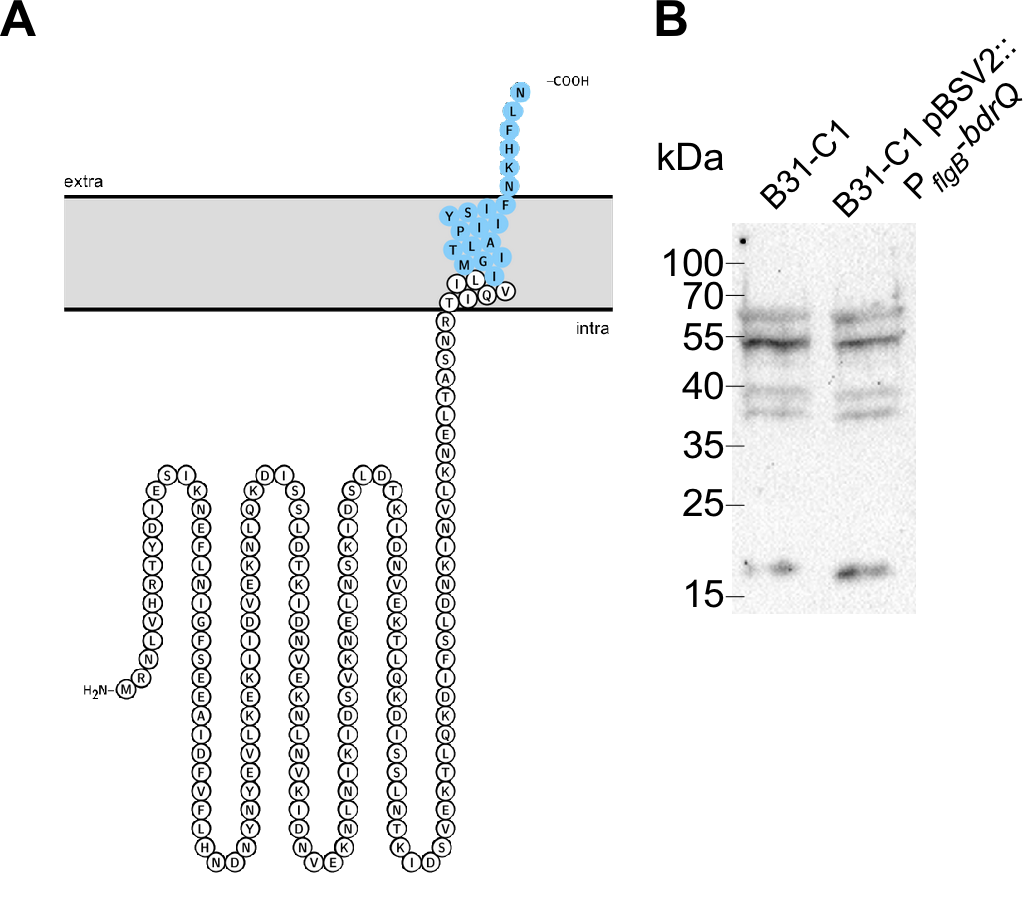

Supplement: Supplementary file 4 [file Image_1.TIF]

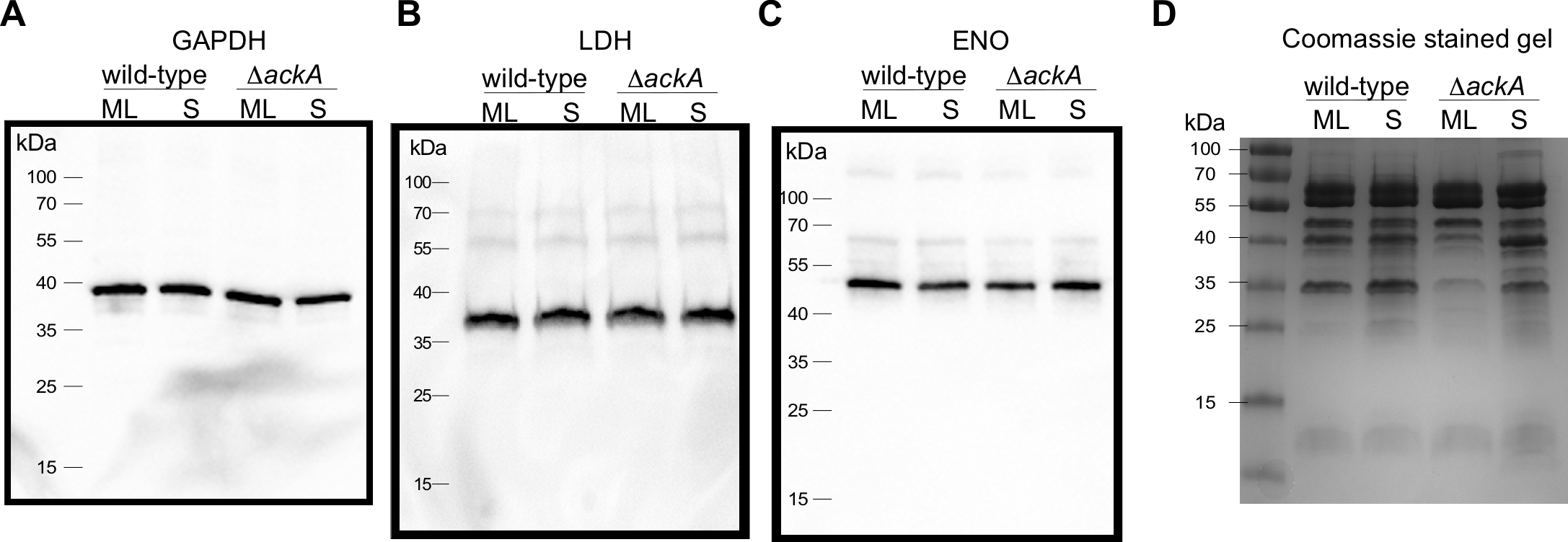

Supplement: Supplementary file 5 [file Image_2.TIF]

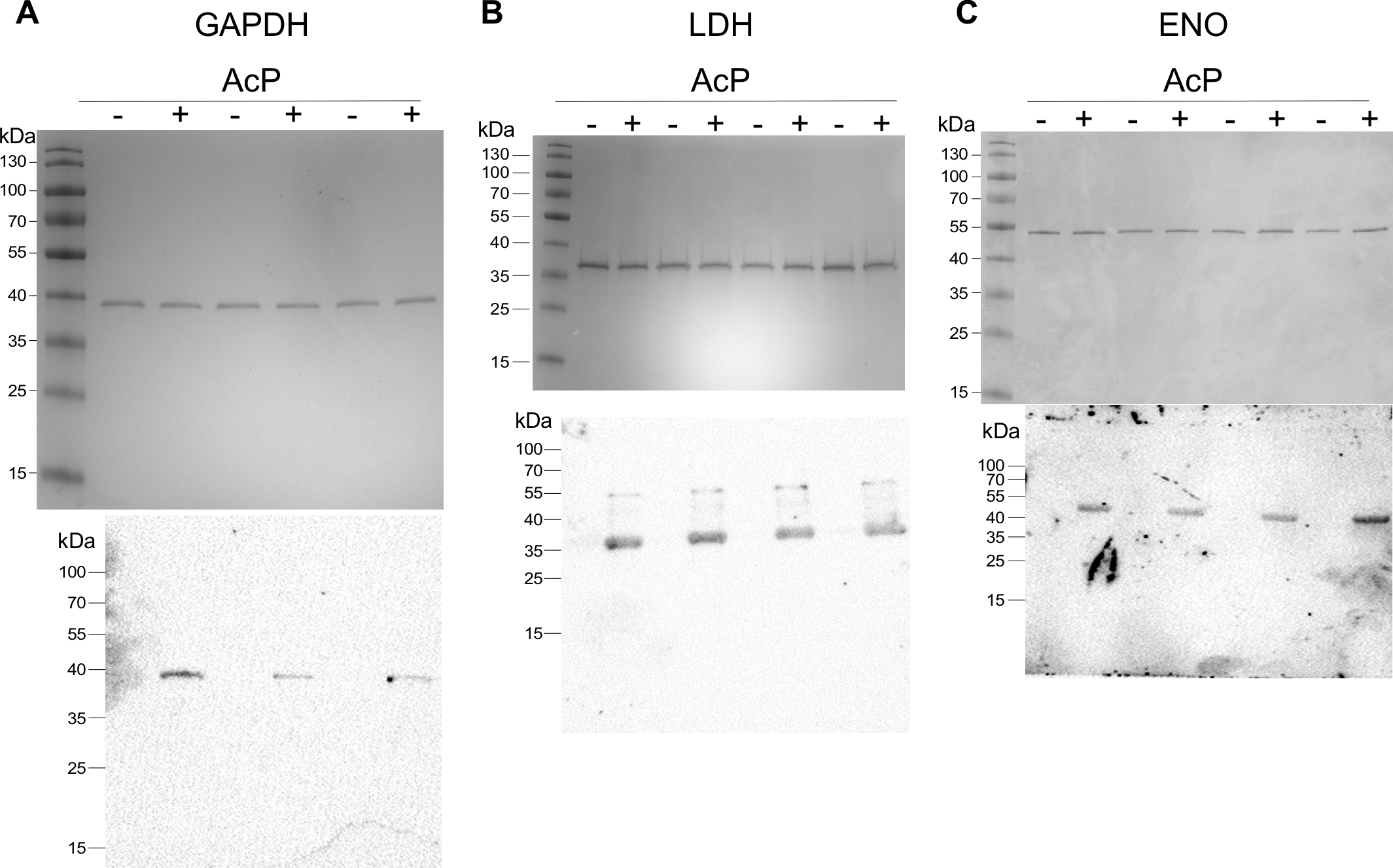

Supplement: Supplementary file 6 [file Image_3.TIF]

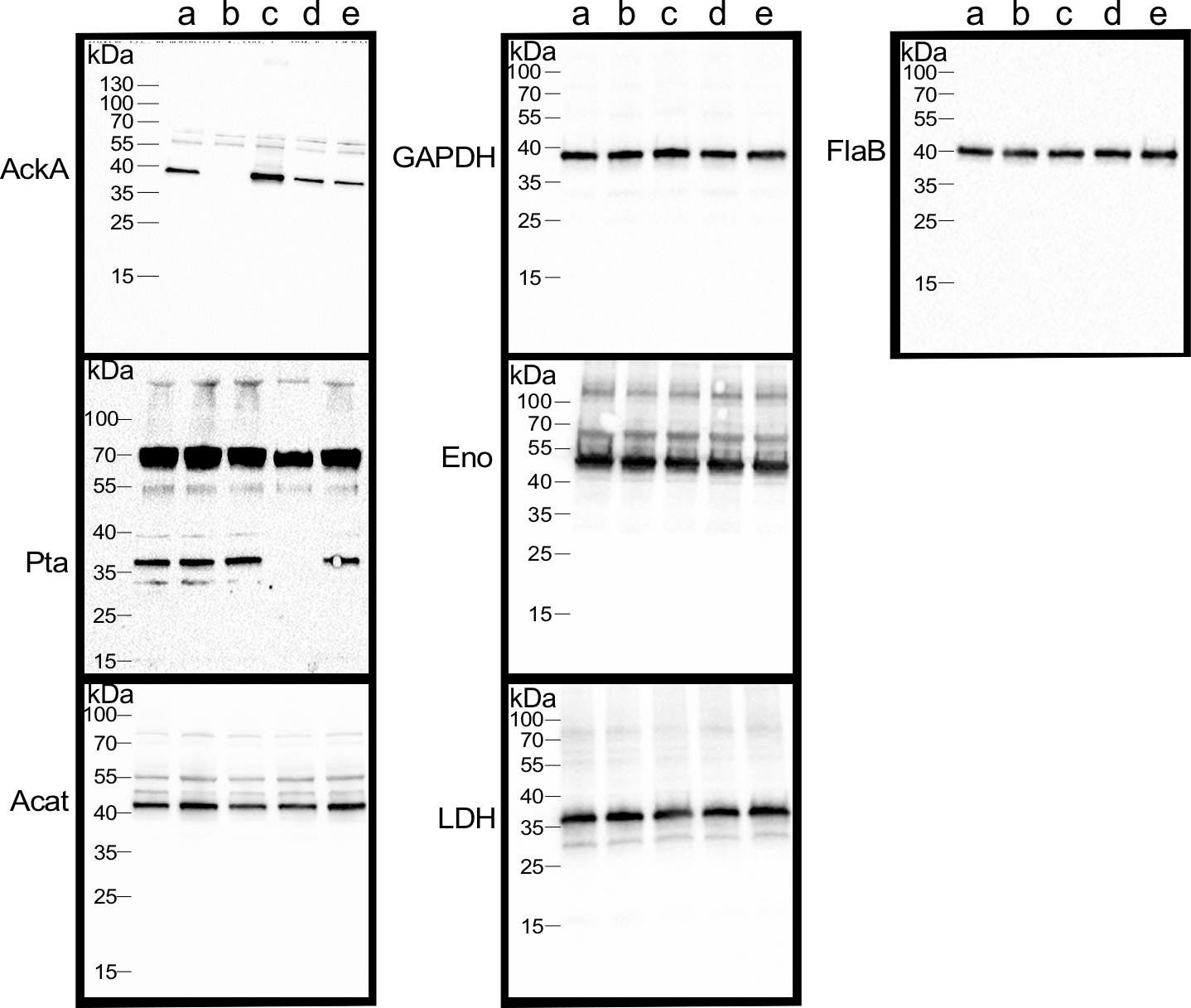

Supplement: Supplementary file 7 [file Image_4.TIF]
