# Supplementary material for: Global Profiling of Lysine Acetylation in Borrelia burgdorferi B31 Reveals Its Role in Central Metabolism
Source: Front Microbiol. 2018 Aug 31;9:2036. doi: 10.3389/fmicb.2018.02036 (PMC6127242; doi:10.3389/fmicb.2018.02036)

Glucose-6-phosphate isomerase - Pgi

NLNELENFk_{ac}ILEGIAPEVLK

Extracted from: D:\DATA_Orbitrap_2016\GALLO_BONTEMPS\GALLO_20160219\Burdo_Kac_stat_4_01.raw #12946 RT: 60.27
ITMS, CID@35.00, z=+3, Mono m/z=775.76080 Da, MH+=2325.26786 Da, Match Tol.=0.35 Da

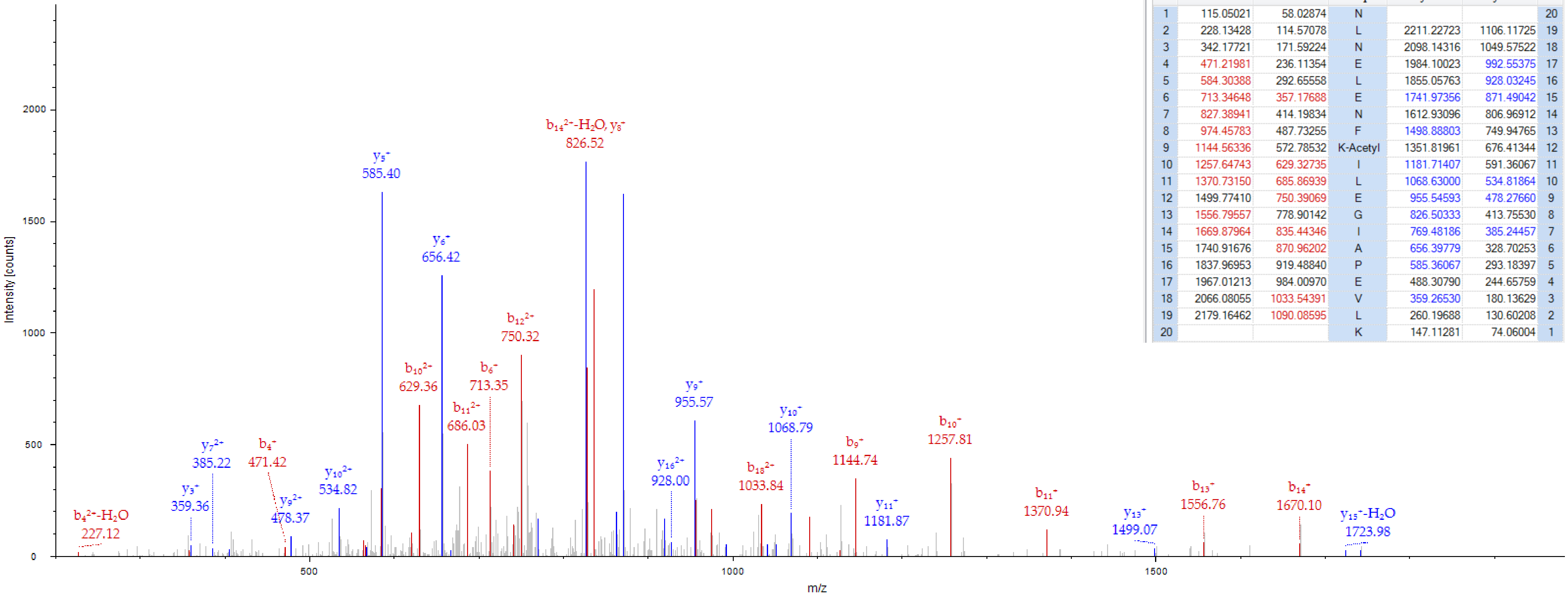

Ion Series | Neutral Losses | Precursor Ions

#1	b⁺	b²⁺	Seq.	y⁺	y²⁺	#2
1	115.05021	58.02874	N			20
2	228.13428	114.57078	L	2211.22723	1106.11725	19
3	342.17721	171.59224	N	2098.14316	1049.57522	18
4	471.21981	236.11354	E	1984.10023	992.55375	17
5	584.30388	292.65558	L	1855.05763	928.03245	16
6	713.34648	357.17688	E	1741.97356	871.49042	15
7	827.38941	414.19834	N	1612.93096	806.96912	14
8	974.45783	487.73255	F	1498.88803	749.94765	13
9	1144.56336	572.78532	K-Acetyl	1351.81961	676.41344	12
10	1257.64743	629.32735	I	1181.71407	591.36067	11
11	1370.73150	685.86939	L	1068.63000	534.81864	10
12	1499.77410	750.39069	E	955.54593	478.27660	9
13	1556.79557	778.90142	G	826.50333	413.75530	8
14	1669.87964	835.44346	I	769.48186	385.24457	7
15	1740.91676	870.96202	A	656.39779	328.70253	6
16	1837.96953	919.48840	P	585.36067	293.18397	5
17	1967.01213	984.00970	E	488.30790	244.65759	4
18	2066.08055	1033.54391	V	359.26530	180.13629	3
19	2179.16462	1090.08595	L	260.19688	130.60208	2
20			K	147.11281	74.06004	1

Glucose-6-phosphate isomerase - Pgi

NDNFk_{ac}DEVIESYSK

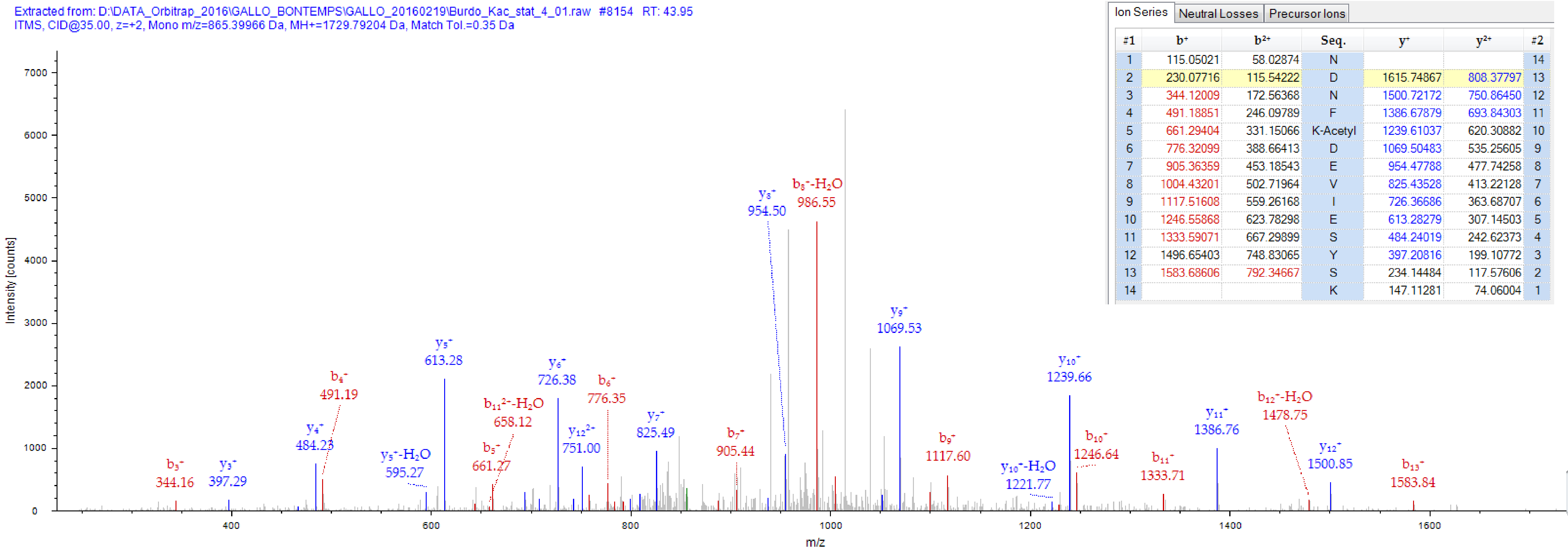

#1	b⁺	b²⁺	Seq.	y⁺	y²⁺	#2
1	115.05021	58.02874	N			14
2	230.07716	115.54222	D	1615.74867	808.37797	13
3	344.12009	172.56368	N	1500.72172	750.86450	12
4	491.18851	246.09789	F	1386.67879	693.84303	11
5	661.29404	331.15066	K-Acetyl	1239.61037	620.30882	10
6	776.32099	388.66413	D	1069.50483	535.25605	9
7	905.36359	453.18543	E	954.47788	477.74258	8
8	1004.43201	502.71964	V	825.43528	413.22128	7
9	1117.51608	559.26168	I	726.36686	363.68707	6
10	1246.55868	623.78298	E	613.28279	307.14503	5
11	1333.59071	667.29899	S	484.24019	242.62373	4
12	1496.65403	748.83065	Y	397.20816	199.10772	3
13	1583.68606	792.34667	S	234.14484	117.57606	2
14			K	147.11281	74.06004	1

Glyceraldehyde-3-phosphate dehydrogenase - GAPDH

EIk$_{ac}$IIAER

Extracted from: D:\DATA_Orbitrap_2016\GALLO_BONTEMPS\GALLO_20160219\Burdo_Kac_stat_4_01.raw #5191 RT: 33.77
ITMS, CID@35.00, z=+2, Mono m/z=507.30249 Da, MH+=1013.59770 Da, Match Tol.=0.35 Da

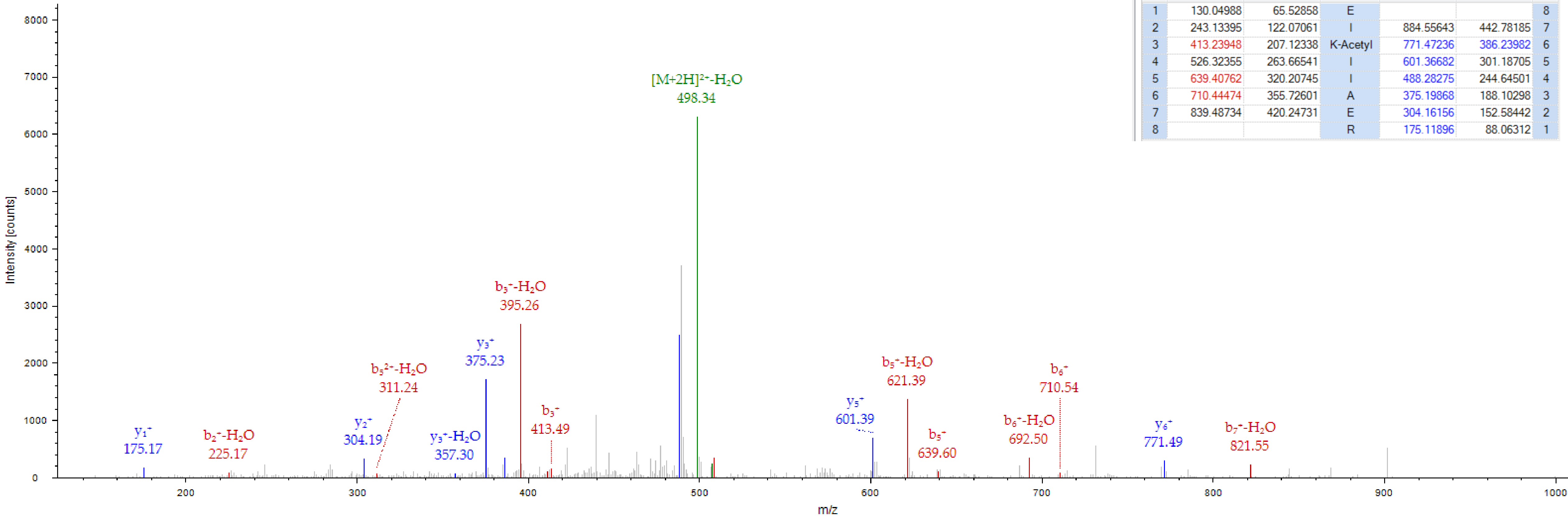

Ion Series | Neutral Losses | Precursor Ions

#1	b^+	b^{2+}	Seq.	y^+	y^{2+}	#2
1	130.04988	65.52858	E			8
2	243.13395	122.07061	I	884.55643	442.78185	7
3	413.23948	207.12338	K-Acetyl	771.47236	386.23982	6
4	526.32355	263.66541	I	601.36682	301.18705	5
5	639.40762	320.20745	I	488.28275	244.64501	4
6	710.44474	355.72601	A	375.19868	188.10298	3
7	839.48734	420.24731	E	304.16156	152.58442	2
8			R	175.11896	88.06312	1

Glyceraldehyde-3-phosphate dehydrogenase - GAPDH

AVGLVLPELk_{ac}GK

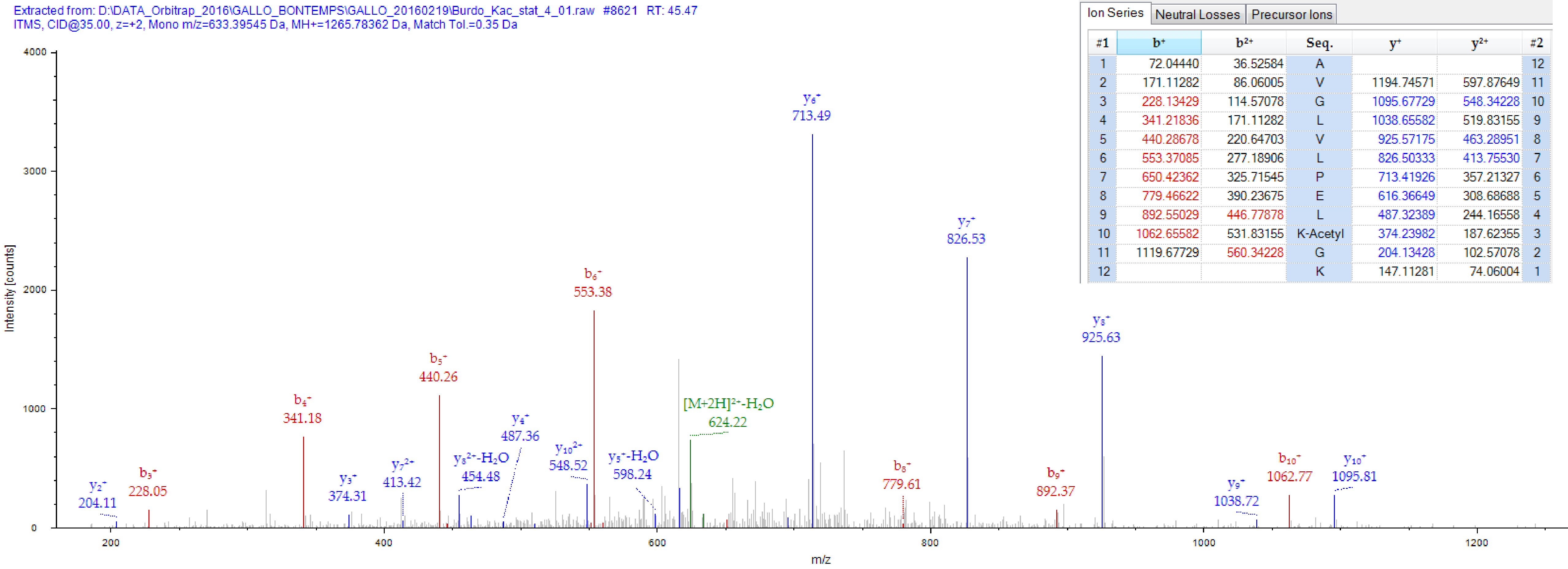

Ion Series | Neutral Losses | Precursor Ions

#1	b+	b2+	Seq.	y+	y2+	#2
1	72.04440	36.52584	A			12
2	171.11282	86.06005	V	1194.74571	597.87649	11
3	228.13429	114.57078	G	1095.67729	548.34228	10
4	341.21836	171.11282	L	1038.65582	519.83155	9
5	440.28678	220.64703	V	925.57175	463.28951	8
6	553.37085	277.18906	L	826.50333	413.75530	7
7	650.42362	325.71545	P	713.41926	357.21327	6
8	779.46622	390.23675	E	616.36649	308.68688	5
9	892.55029	446.77878	L	487.32389	244.16558	4
10	1062.65582	531.83155	K-Acetyl	374.23982	187.62355	3
11	1119.67729	560.34228	G	204.13428	102.57078	2
12			K	147.11281	74.06004	1

Glyceraldehyde-3-phosphate dehydrogenase - GAPDH

DVTk$_{ac}$EEINSVLR

Extracted from: D:\DATA_Orbitrap_2016\GALLO_BONTEMPS\GALLO_20160219\Burdo_Kac_stat_4_01.raw #7798 RT: 42.79
ITMS, CID@35.00, z=+2, Mono m/z=722.88464 Da, MH+=1444.76201 Da, Match Tol.=0.35 Da

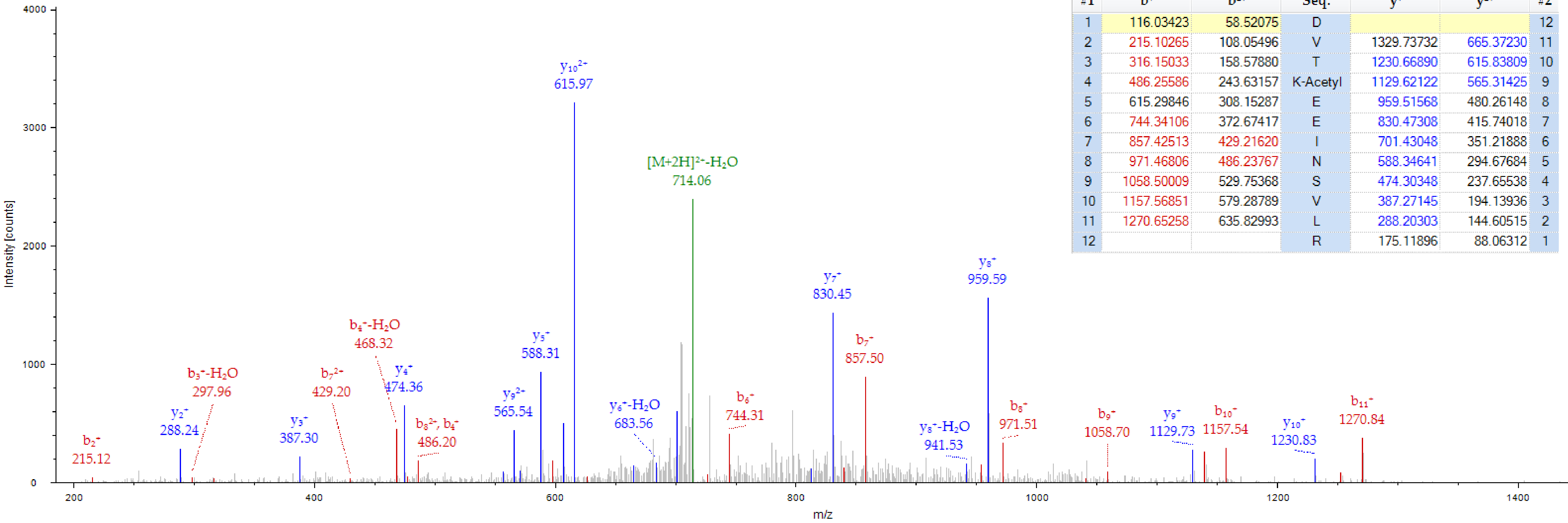

Ion Series | Neutral Losses | Precursor Ions

#1	b+	b2+	Seq.	y+	y2+	#2
1	116.03423	58.52075	D			12
2	215.10265	108.05496	V	1329.73732	665.37230	11
3	316.15033	158.57880	T	1230.66890	615.83809	10
4	486.25586	243.63157	K-Acetyl	1129.62122	565.31425	9
5	615.29846	308.15287	E	959.51568	480.26148	8
6	744.34106	372.67417	E	830.47308	415.74018	7
7	857.42513	429.21620	I	701.43048	351.21888	6
8	971.46806	486.23767	N	588.34641	294.67684	5
9	1058.50009	529.75368	S	474.30348	237.65538	4
10	1157.56851	579.28789	V	387.27145	194.13936	3
11	1270.65258	635.82993	L	288.20303	144.60515	2
12			R	175.11896	88.06312	1

Glyceraldehyde-3-phosphate dehydrogenase - GAPDH

VVDLAQk_{ac}LVK

Extracted from: D:\DATA_Orbitrap_2016\GALLO_BONTEMPS\GALLO_20160208\Burdo_Kac_stat__sansNP40_01.raw #8243 RT: 42.76
ITMS, CID@35.00, z=+2, Mono m/z=577.86023 Da, MH+=1154.71318 Da, Match Tol.=0.35 Da

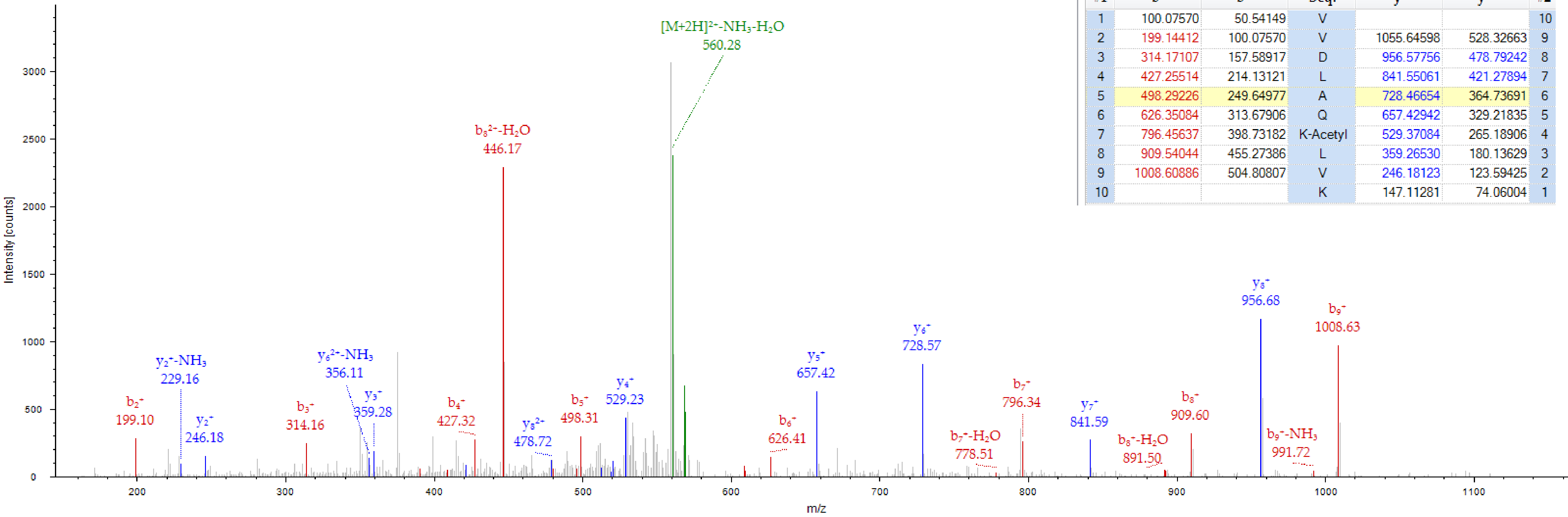

Ion Series | Neutral Losses | Precursor Ions

#1	b⁺	b²⁺	Seq.	y⁺	y²⁺	#2
1	100.07570	50.54149	V			10
2	199.14412	100.07570	V	1055.64598	528.32663	9
3	314.17107	157.58917	D	956.57756	478.79242	8
4	427.25514	214.13121	L	841.55061	421.27894	7
5	498.29226	249.64977	A	728.46654	364.73691	6
6	626.35084	313.67906	Q	657.42942	329.21835	5
7	796.45637	398.73182	K-Acetyl	529.37084	265.18906	4
8	909.54044	455.27386	L	359.26530	180.13629	3
9	1008.60886	504.80807	V	246.18123	123.59425	2
10			K	147.11281	74.06004	1

2,3-bisphosphoglycerate-dependent phosphoglycerate mutase - GpmA

Ion Series	Neutral Losses	Precursor Ions				
#1	b^+	b^{2+}	Seq.	y^+	y^{2+}	#2
1	138.06619	69.53673	H			11
2	301.12951	151.06839	Y	1257.63618	629.32173	10
3	464.19283	232.60005	Y	1094.57286	547.79007	9
4	577.27690	289.14209	L	931.50954	466.25841	8
5	634.29837	317.65282	G	818.42547	409.71637	7
6	749.32532	375.16630	D	761.40400	381.20564	6
7	878.36792	439.68760	E	646.37705	323.69216	5
8	965.39995	483.20361	S	517.33445	259.17086	4
9	1135.50548	568.25638	K-Acetyl	430.30242	215.65485	3
10	1248.58955	624.79841	I	260.19688	130.60208	2
11			K	147.11281	74.06004	1

HYYLGDESk_{ac}IK

Extracted from: D:\DATA_Orbitrap_2016\GALLO_BONTEMPS\GALLO_20160219\Burdo_Kac_stat_4_01.raw #4396 RT: 30.62
ITMS, CID@35.00, z=+2, Mono m/z=697.85278 Da, MH+=1394.69829 Da, Match Tol.=0.35 Da

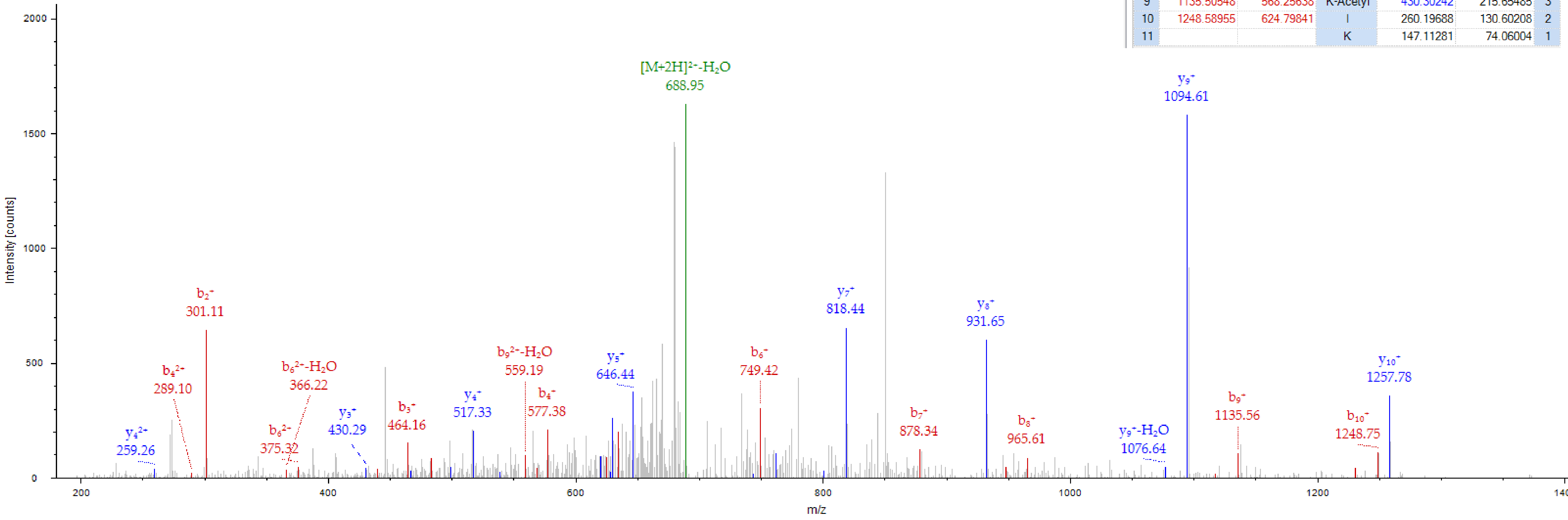

2,3-bisphosphoglycerate-dependent phosphoglycerate mutase - GpmA

AMESVASQGk_{ac}LK

Extracted from: D:\DATA_Orbitrap_2016\GALLO_BONTEMPS\GALLO_20160219\Burdo_Kac_stat_4_01.raw #3826 RT: 28.24
ITMS, CID@35.00, z=+2, Mono m/z=645.84027 Da, MH+=1290.67327 Da, Match Tol.=0.35 Da

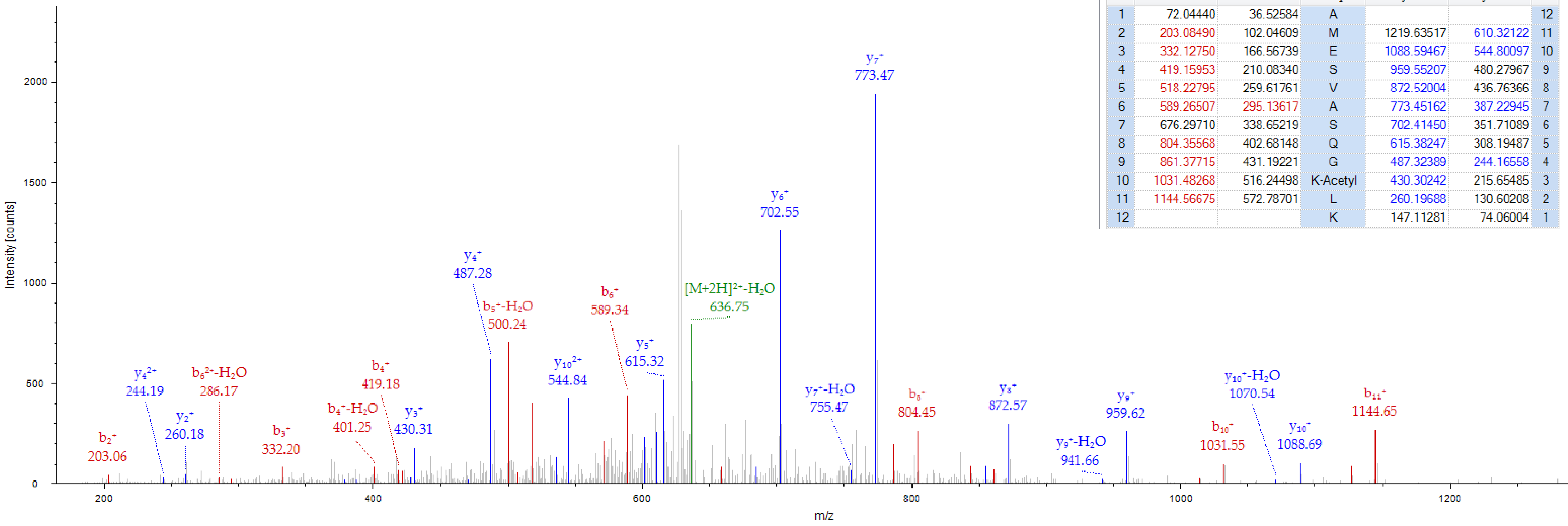

Ion Series | Neutral Losses | Precursor Ions

#1	b^+	b^{2+}	Seq.	y^+	y^{2+}	#2
1	72.04440	36.52584	A			12
2	203.08490	102.04609	M	1219.63517	610.32122	11
3	332.12750	166.56739	E	1088.59467	544.80097	10
4	419.15953	210.08340	S	959.55207	480.27967	9
5	518.22795	259.61761	V	872.52004	436.76366	8
6	589.26507	295.13617	A	773.45162	387.22945	7
7	676.29710	338.65219	S	702.41450	351.71089	6
8	804.35568	402.68148	Q	615.38247	308.19487	5
9	861.37715	431.19221	G	487.32389	244.16558	4
10	1031.48268	516.24498	K-Acetyl	430.30242	215.65485	3
11	1144.56675	572.78701	L	260.19688	130.60208	2
12			K	147.11281	74.06004	1

Enolase - Eno

YVLk$_{ac}$WSTK

Ion Series | Neutral Losses | Precursor Ions

#1	b⁺	b²⁺	Seq.	y⁺	y²⁺	#2
1	164.07060	82.53894	Y			8
2	263.13902	132.07315	V	903.52987	452.26857	7
3	376.22309	188.61518	L	804.46145	402.73436	6
4	546.32862	273.66795	K-Acetyl	691.37738	346.19233	5
5	732.40794	366.70761	W	521.27184	261.13956	4
6	819.43997	410.22362	S	335.19252	168.09990	3
7	920.48765	460.74746	T	248.16049	124.58388	2
8			K	147.11281	74.06004	1

Extracted from: D:\DATA_Orbitrap_2016\GALLO_BONTEMPS\GALLO_20160219\Burdo_Kac_stat_4_01.raw #6473 RT: 38.27
ITMS, CID@35.00, z=+2, Mono m/z=533.80011 Da, MH+=1066.59294 Da, Match Tol.=0.35 Da

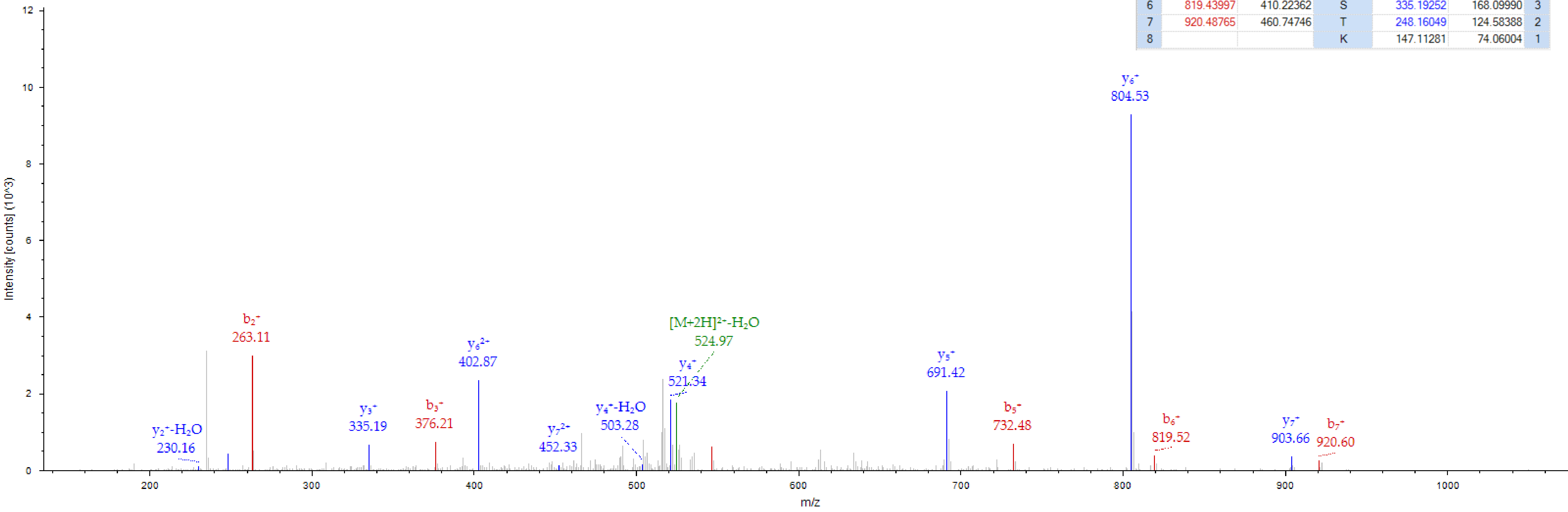

Enolase - Eno

LTSEQMVEYWAk_{ac}WVEK

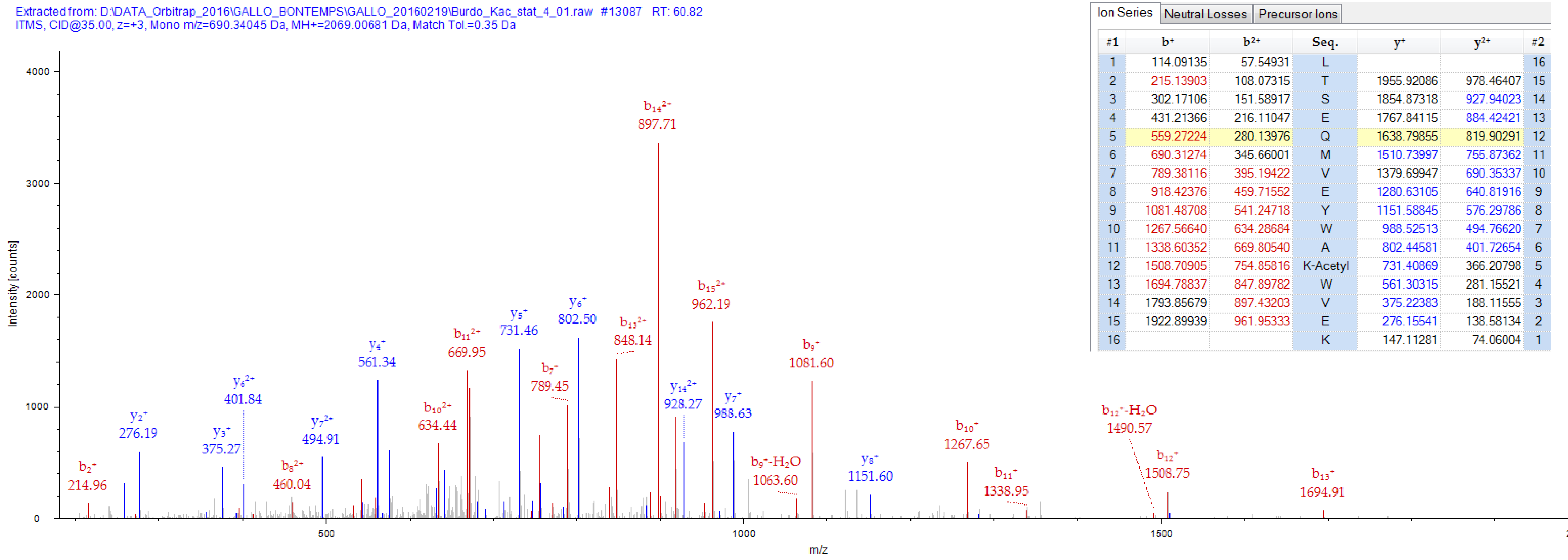

Ion Series | Neutral Losses | Precursor Ions

#1	b^+	b^{2+}	Seq.	y^+	y^{2+}	#2
1	114.09135	57.54931	L			16
2	215.13903	108.07315	T	1955.92086	978.46407	15
3	302.17106	151.58917	S	1854.87318	927.94023	14
4	431.21366	216.11047	E	1767.84115	884.42421	13
5	559.27224	280.13976	Q	1638.79855	819.90291	12
6	690.31274	345.66001	M	1510.73997	755.87362	11
7	789.38116	395.19422	V	1379.69947	690.35337	10
8	918.42376	459.71552	E	1280.63105	640.81916	9
9	1081.48708	541.24718	Y	1151.58845	576.29786	8
10	1267.56640	634.28684	W	988.52513	494.76620	7
11	1338.60352	669.80540	A	802.44581	401.72654	6
12	1508.70905	754.85816	K-Acetyl	731.40869	366.20798	5
13	1694.78837	847.89782	W	561.30315	281.15521	4
14	1793.85679	897.43203	V	375.22383	188.11555	3
15	1922.89939	961.95333	E	276.15541	138.58134	2
16			K	147.11281	74.06004	1

Enolase - Eno

IGNk_{ac}IQLVGDDLFVTNTSFLK

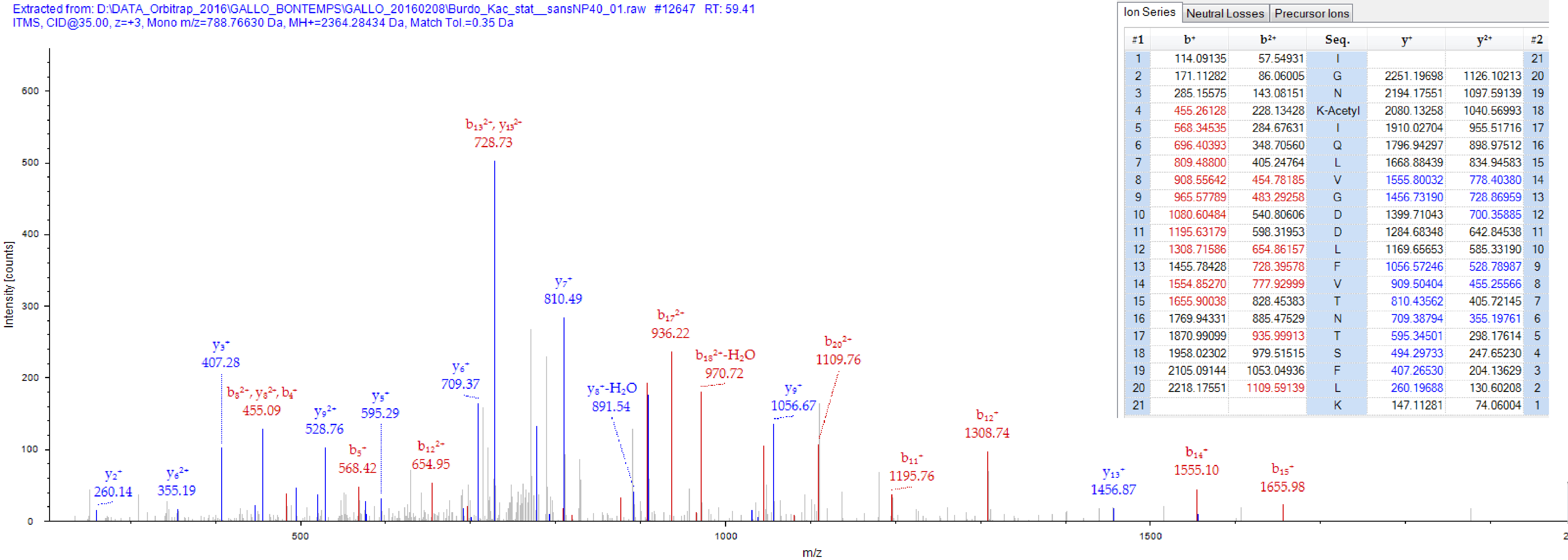

#1	b⁺	b²⁺	Seq.	y⁺	y²⁺	#2
1	114.09135	57.54931	I			21
2	171.11282	86.06005	G	2251.19698	1126.10213	20
3	285.15575	143.08151	N	2194.17551	1097.59139	19
4	455.26128	228.13428	K-Acetyl	2080.13258	1040.56993	18
5	568.34535	284.67631	I	1910.02704	955.51716	17
6	696.40393	348.70560	Q	1796.94297	898.97512	16
7	809.48800	405.24764	L	1668.88439	834.94583	15
8	908.55642	454.78185	V	1555.80032	778.40380	14
9	965.57789	483.29258	G	1456.73190	728.86959	13
10	1080.60484	540.80606	D	1399.71043	700.35885	12
11	1195.63179	598.31953	D	1284.68348	642.84538	11
12	1308.71586	654.86157	L	1169.65653	585.33190	10
13	1455.78428	728.39578	F	1056.57246	528.78987	9
14	1554.85270	777.92999	V	909.50404	455.25566	8
15	1655.90038	828.45383	T	810.43562	405.72145	7
16	1769.94331	885.47529	N	709.38794	355.19761	6
17	1870.99099	935.99913	T	595.34501	298.17614	5
18	1958.02302	979.51515	S	494.29733	247.65230	4
19	2105.09144	1053.04936	F	407.26530	204.13629	3
20	2218.17551	1109.59139	L	260.19688	130.60208	2
21			K	147.11281	74.06004	1

L-lactate dehydrogenase - LDH

AKGEVMDLNHGQMFLk_{ac}K

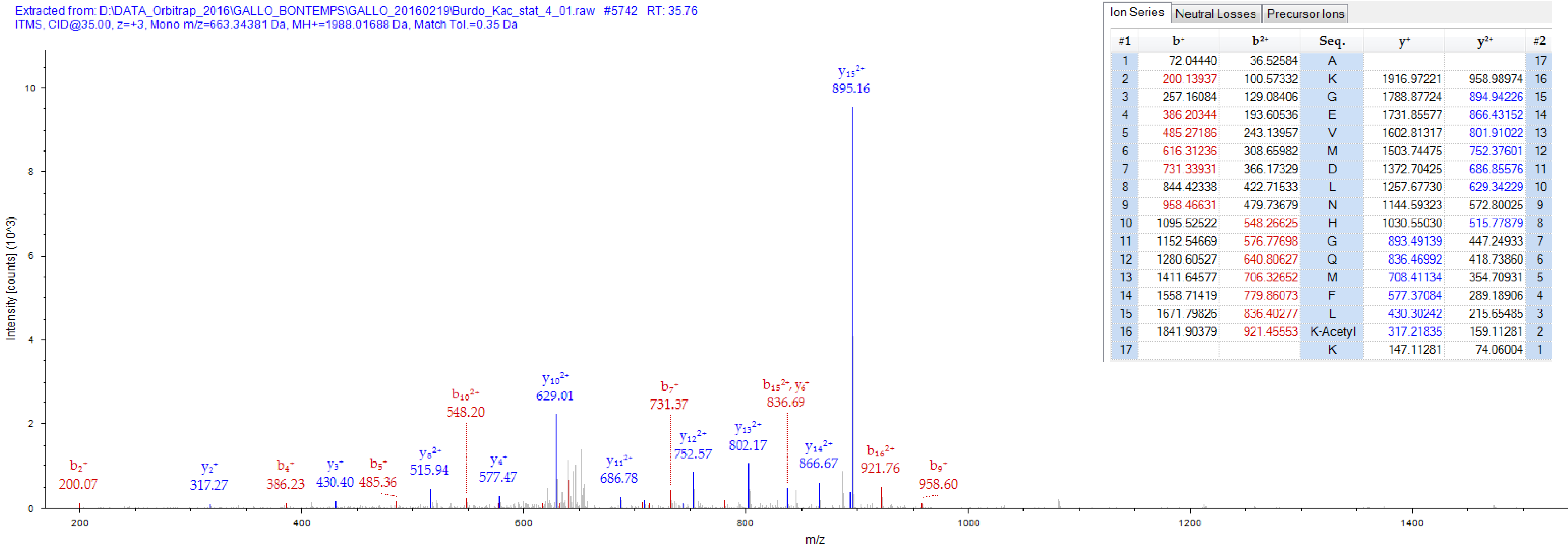

Ion Series | Neutral Losses | Precursor Ions

#1	b^+	b^{2+}	Seq.	y^+	y^{2+}	#2
1	72.04440	36.52584	A			17
2	200.13937	100.57332	K	1916.97221	958.98974	16
3	257.16084	129.08406	G	1788.87724	894.94226	15
4	386.20344	193.60536	E	1731.85577	866.43152	14
5	485.27186	243.13957	V	1602.81317	801.91022	13
6	616.31236	308.65982	M	1503.74475	752.37601	12
7	731.33931	366.17329	D	1372.70425	686.85576	11
8	844.42338	422.71533	L	1257.67730	629.34229	10
9	958.46631	479.73679	N	1144.59323	572.80025	9
10	1095.52522	548.26625	H	1030.55030	515.77879	8
11	1152.54669	576.77698	G	893.49139	447.24933	7
12	1280.60527	640.80627	Q	836.46992	418.73860	6
13	1411.64577	706.32652	M	708.41134	354.70931	5
14	1558.71419	779.86073	F	577.37084	289.18906	4
15	1671.79826	836.40277	L	430.30242	215.65485	3
16	1841.90379	921.45553	K-Acetyl	317.21835	159.11281	2
17			K	147.11281	74.06004	1

L-lactate dehydrogenase - LDH

LDLVDk_{ac}NSK

Extracted from: D:\DATA_Orbitrap_2016\GALLO_BONTEMPS\GALLO_20160219\Burdo_Kac_stat_4_01.raw #4466 RT: 30.90
ITMS, CID@35.00, z=+2, Mono m/z=537.29608 Da, MH+=1073.58489 Da, Match Tol.=0.35 Da

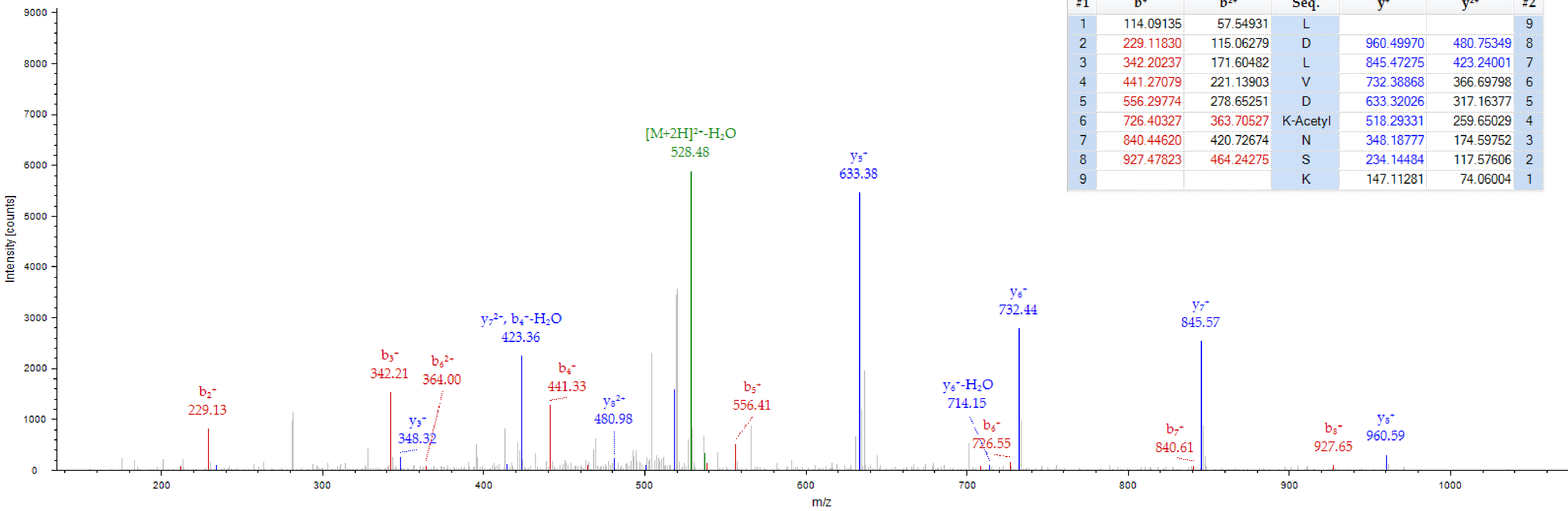

Ion Series | Neutral Losses | Precursor Ions

#1	b⁺	b²⁺	Seq.	y⁺	y²⁺	#2
1	114.09135	57.54931	L			9
2	229.11830	115.06279	D	960.49970	480.75349	8
3	342.20237	171.60482	L	845.47275	423.24001	7
4	441.27079	221.13903	V	732.38868	366.69798	6
5	556.29774	278.65251	D	633.32026	317.16377	5
6	726.40327	363.70527	K-Acetyl	518.29331	259.65029	4
7	840.44620	420.72674	N	348.18777	174.59752	3
8	927.47823	464.24275	S	234.14484	117.57606	2
9			K	147.11281	74.06004	1

L-lactate dehydrogenase - LDH

FNSSANQLk_{ac}SYIDK

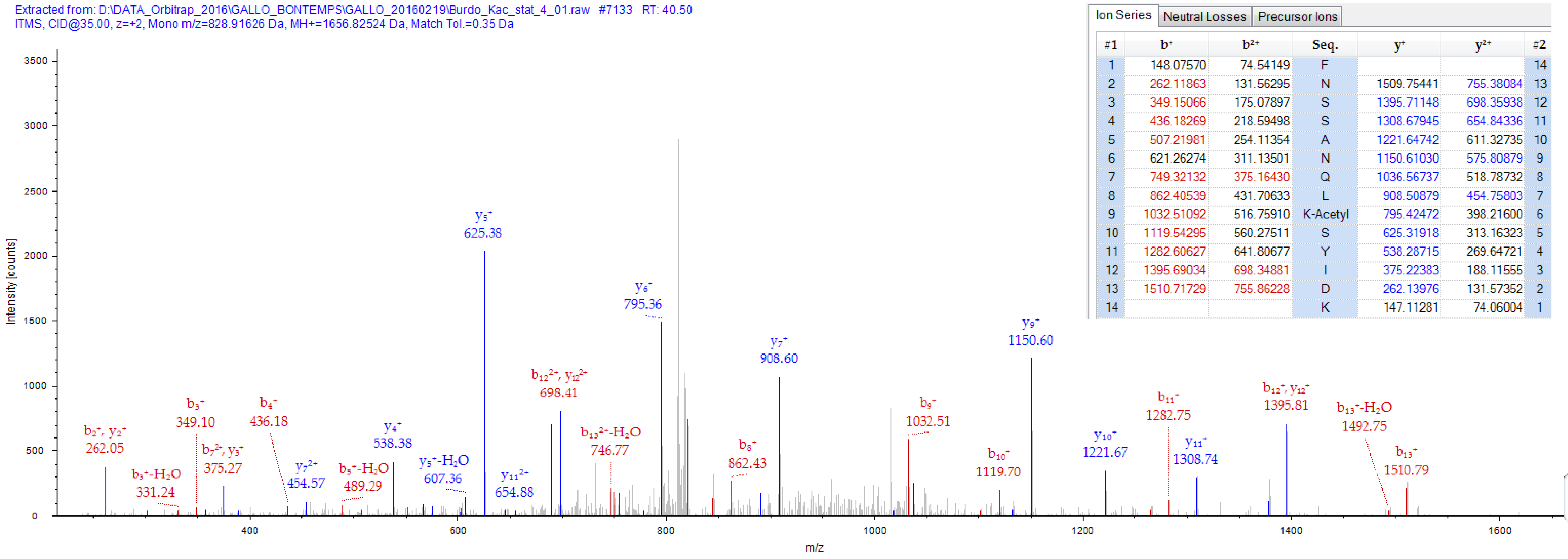

Ion Series | Neutral Losses | Precursor Ions

#1	b⁺	b²⁺	Seq.	y⁺	y²⁺	#2
1	148.07570	74.54149	F			14
2	262.11863	131.56295	N	1509.75441	755.38084	13
3	349.15066	175.07897	S	1395.71148	698.35938	12
4	436.18269	218.59498	S	1308.67945	654.84336	11
5	507.21981	254.11354	A	1221.64742	611.32735	10
6	621.26274	311.13501	N	1150.61030	575.80879	9
7	749.32132	375.16430	Q	1036.56737	518.78732	8
8	862.40539	431.70633	L	908.50879	454.75803	7
9	1032.51092	516.75910	K-Acetyl	795.42472	398.21600	6
10	1119.54295	560.27511	S	625.31918	313.16323	5
11	1282.60627	641.80677	Y	538.28715	269.64721	4
12	1395.69034	698.34881	I	375.22383	188.11555	3
13	1510.71729	755.86228	D	262.13976	131.57352	2
14			K	147.11281	74.06004	1

Triosephosphate isomerase - TpiA

LEITk$_{ac}$LYSK

Extracted from: D:\DATA_Orbitrap_2016\GALLO_BONTEMPS\GALLO_20160219\Burdo_Kac_stat_4_01.raw #6627 RT: 38.83
ITMS, CID@35.00, z=+2, Mono m/z=568.83148 Da, MH+=1136.65569 Da, Match Tol.=0.35 Da

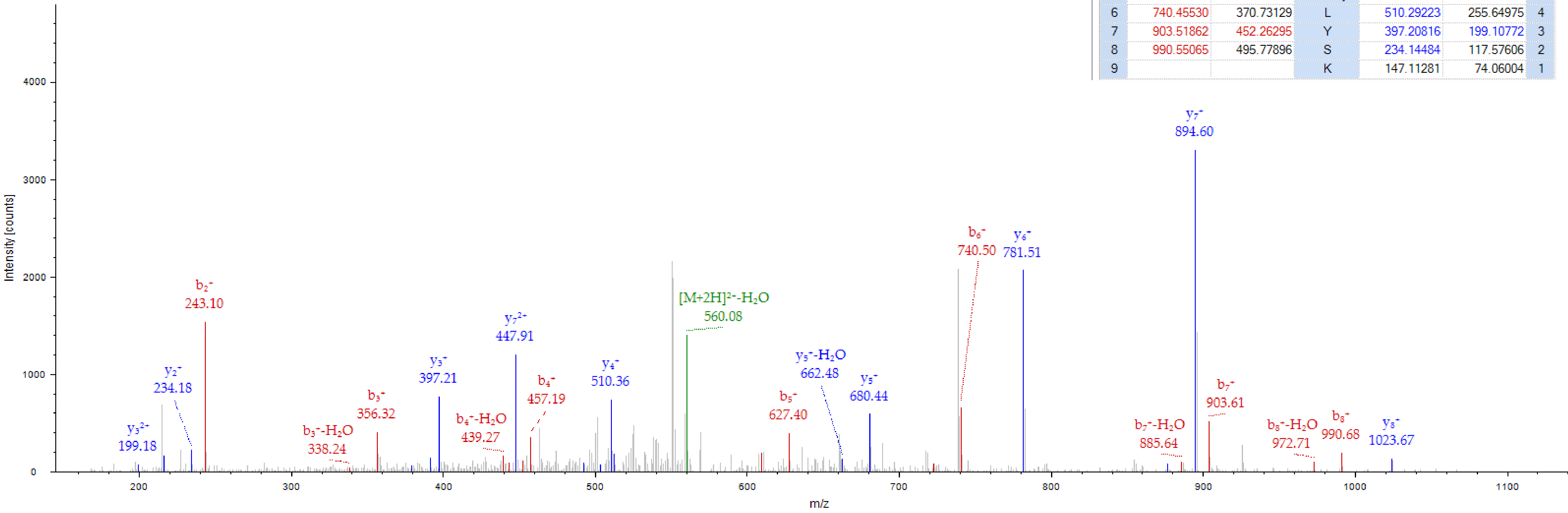

Ion Series | Neutral Losses | Precursor Ions

#1	b⁺	b²⁺	Seq.	y⁺	y²⁺	#2
1	114.09135	57.54931	L			9
2	243.13395	122.07061	E	1023.57212	512.28970	8
3	356.21802	178.61265	I	894.52952	447.76840	7
4	457.26570	229.13649	T	781.44545	391.22636	6
5	627.37123	314.18925	K-Acetyl	680.39777	340.70252	5
6	740.45530	370.73129	L	510.29223	255.64975	4
7	903.51862	452.26295	Y	397.20816	199.10772	3
8	990.55065	495.77896	S	234.14484	117.57606	2
9			K	147.11281	74.06004	1

Triosephosphate isomerase - TpiA

Ion Series	Neutral Losses	Precursor Ions			

#1	b+	b2+	Seq.	y+	y2+	#2
1	114.09135	57.54931	L			24
2	277.15467	139.08097	Y	2500.23150	1250.61939	23
3	364.18670	182.59699	S	2337.16818	1169.08773	22
4	534.29223	267.64975	K-Acetyl	2250.13615	1125.57171	21
5	621.32426	311.16577	S	2080.03061	1040.51894	20
6	692.36138	346.68433	A	1992.99858	997.00293	19
7	779.39341	390.20034	S	1921.96146	961.48437	18
8	894.42036	447.71382	D	1834.92943	917.96835	17
9	1008.46329	504.73528	N	1719.90248	860.45488	16
10	1121.54736	561.27732	I	1605.85955	803.43341	15
11	1234.63143	617.81935	I	1492.77548	746.89138	14
12	1347.71550	674.36139	I	1379.69141	690.34934	13
13	1475.77408	738.39068	Q	1266.60734	633.80731	12
14	1638.83740	819.92234	Y	1138.54876	569.77802	11
15	1695.85887	848.43307	G	975.48544	488.24636	10
16	1752.88034	876.94381	G	918.46397	459.73562	9
17	1839.91237	920.45982	S	861.44250	431.22489	8
18	1938.98079	969.99403	V	774.41047	387.70887	7
19	2053.02372	1027.01550	N	675.34205	338.17466	6
20	2140.05575	1070.53151	S	561.29912	281.15320	5
21	2254.09868	1127.55298	N	474.26709	237.63718	4
22	2368.14161	1184.57444	N	360.22416	180.61572	3
23	2467.21003	1234.10865	V	246.18123	123.59425	2
24			K	147.11281	74.06004	1

LYSk$_{ac}$SASDNIIIQYGGSVNSNNVK

Extracted from: D:\DATA_Orbitrap_2016\GALLO_BONTEMPS\GALLO_20160208\Burdo_Kac_stat__sansNP40_01.raw #8274 RT: 42.88
ITMS, CID@35.00, z=+2, Mono m/z=1307.16406 Da, MH+=2613.32085 Da, Match Tol.=0.35 Da

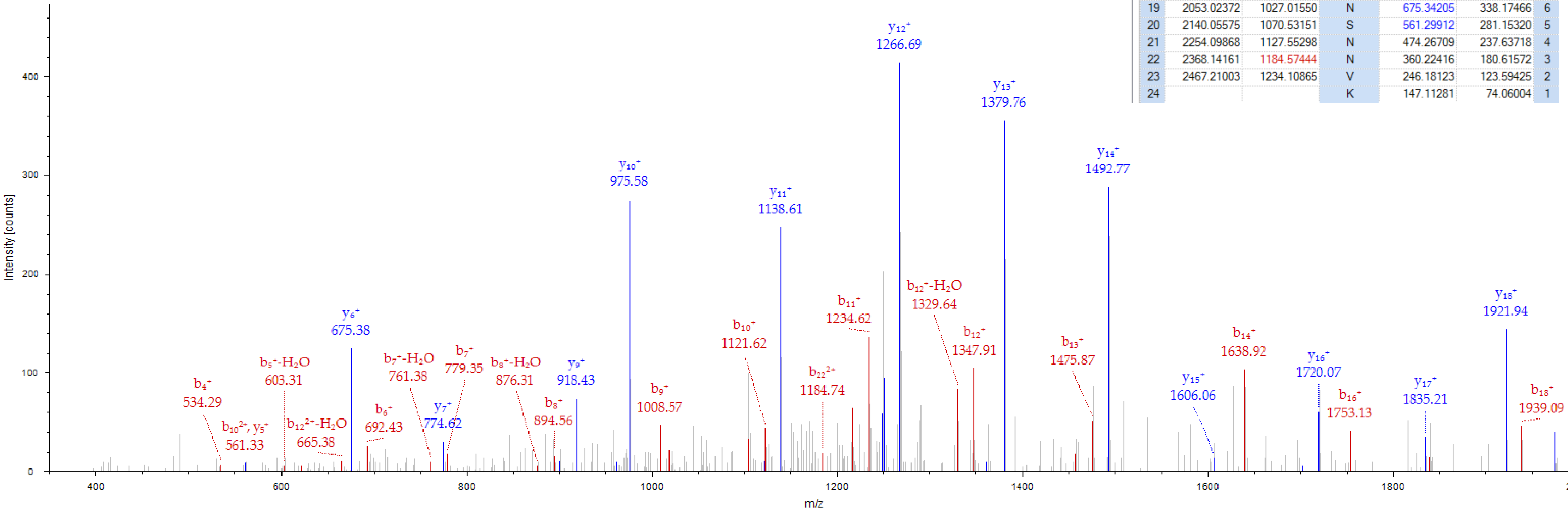

Supplement: Supplementary file 8 [file Image_5.pdf]
